# Integrated Genome-Scale Prediction of Detrimental Mutations in Transcription Networks

**DOI:** 10.1371/journal.pgen.1002077

**Published:** 2011-05-26

**Authors:** Mirko Francesconi, Rob Jelier, Ben Lehner

**Affiliations:** 1 EMBL-CRG Systems Biology Research Unit, Centre for Genomic Regulation, Universitat Pompeu Fabra, Barcelona Spain; 2 Dottorato di Ricerca in "Biotecnologie Cellulari e Molecolari," Dipartimento di Biochimica "G. Moruzzi," Università di Bologna, Bologna, Italy; 3 Institució Catalana de Recerca i Estudis Avançats, Centre for Genomic Regulation, Universitat Pompeu Fabra, Barcelona, Spain; University of Washington, United States of America

## Abstract

A central challenge in genetics is to understand when and why mutations alter the phenotype of an organism. The consequences of gene inhibition have been systematically studied and can be predicted reasonably well across a genome. However, many sequence variants important for disease and evolution may alter gene regulation rather than gene function. The consequences of altering a regulatory interaction (or “edge”) rather than a gene (or “node”) in a network have not been as extensively studied. Here we use an integrative analysis and evolutionary conservation to identify features that predict when the loss of a regulatory interaction is detrimental in the extensively mapped transcription network of budding yeast. Properties such as the strength of an interaction, location and context in a promoter, regulator and target gene importance, and the potential for compensation (redundancy) associate to some extent with interaction importance. Combined, however, these features predict quite well whether the loss of a regulatory interaction is detrimental across many promoters and for many different transcription factors. Thus, despite the potential for regulatory diversity, common principles can be used to understand and predict when changes in regulation are most harmful to an organism.

## Author Summary

The genomes of individuals differ in sequence at thousands of base pairs. Some of these polymorphisms affect the sequence of proteins, but many are likely to alter how genes are regulated. When are changes in gene regulation detrimental to an organism? We have used an integrative analysis of transcription factor binding site conservation in budding yeast to address the extent to which different features predict when potential changes in gene regulation are detrimental. We found that, despite the diversity of transcription factors and regulatory regions in a genome, a few simple properties can be used to predict and understand when changes in regulation are most harmful.

## Introduction

An important challenge in genetics is to understand when and why mutations affect the phenotype of an organism, and when and why they do not. Mutations in protein coding sequences have been extensively studied and loss-of-function phenotypes can be predicted with reasonable accuracy across entire genomes [1], [2]. However, many sequence polymorphisms within a species, and many changes between species lie outside of protein coding regions. These sequence changes will not alter the function of genes themselves, but have the potential to alter the regulatory interactions among genes [3]–[6]. Changes in regulatory regions have been suggested to underlie many phenotypic differences between species [7]–[9] and may account for many disease-causing mutations in humans [10]. Mutations within proteins that influence protein-protein interactions have been termed ‘edgetic’ perturbations [11], [12]. Similarly, mutations in regulatory regions can be considered as altering an ‘edge’ in a regulatory network that connects genes.

One of the most important types of interaction in a cell is mediated via the binding of transcription factors (TFs) to DNA. TFs typically recognize short and degenerate target sequences [13] that occur at high frequency in large eukaryotic genomes [14]. Genome-wide localization analyses using chromatin immunoprecipitation confirm that most TFs indeed associate with hundreds or thousands of sites in a genome [15]–[21].

Not all binding sites for a TF will, however, be of equal functional importance. Whereas the removal of some sites may reduce the fitness of an organism, other sites may change without any phenotypic effect. The constraints on the sequence of a transcription factor binding site are quite well understood, relating to the contribution of a position within the site to the overall binding score [22]. However, properties that associate with differences in functional importance among sites are less clear. Previous studies have attempted to correlate changes in binding sites to changes in gene expression, but this approach has only been informative for a subset of genes [23]–[25].

Here we address the question of whether using a few basic features it is possible to predict when the loss of a binding site is detrimental to an organism. Are there functional properties that characterize the binding sites most important for fitness? Or does the diversity of TFs and regulatory possibilities preclude such an analysis? We use the transcription regulatory network of budding yeast as a model system and evolutionary conservation to identify functionally important interactions. We rely on the assumption that, unless there is functional compensation, binding site losses detrimental to fitness will be purged by purifying selection. We analyze the association and independence of both previously suggested [26]–[30] and novel features with binding site conservation. We then show that with a combination of features we can predict binding site conservation reasonably well across the genome. Informative features include the context of a promoter, the potential for redundancy among sites and among different TFs, the importance of the TF and the target gene, the location of a site in the promoter and genome, and the strength of a binding site. Importantly, these are relatively general properties, because they predict similarly well binding site conservation for many different individual TFs across all of the promoters in the genome. Thus, despite the potential for complexity and diversity, a limited number of principles can be used to understand the importance of mutations that perturb interactions rather than genes in a network.

## Results

### Defining TF binding site and interaction conservation within and between species

We focused our analysis on transcription factor (TF) binding sites defined from large-scale chromatin immunoprecipitation analyses in *Saccharomyces cerevisiae* [16], [31]. This dataset consists of 19,671 binding sites for 119 different TFS in the promoter regions of 3,832 genes and defines 12,012 potential transcription interactions (or ’edges‚ in a network – an edge being defined if at least one binding site for a transcription factor (TF) is present in a promoter). To identify binding sites and interactions that are important for fitness we analyzed their conservation within and between species. Our assumption is that detrimental changes in binding sites will be purged by natural selection. We analyzed the conservation of binding sites both within and between species. The effects of selection should be more apparent between species [32], and the results presented below are consistent with this.

Throughout most of this manuscript we consider a binding site as functionally conserved if its binding score assessed using a position specific scoring matrix (PSSM) is at least 60% of the optimum for that TF, as in Harbison et al. [16]. However, as shown below and in the supplementary material, our conclusions do not depend upon this use of a hard threshold to define functional conservation. We consider a transcription interaction as conserved if at least one binding site for a particular TF is found anywhere in the promoter of a target gene. Binding site conservation within species was determined using the complete genome sequences of 36 natural isolate strains of *S. cerevisiae* [33]. For the experimentally defined sites, 89% are identical in sequence across all strains and 92% are considered as functionally conserved with at least 96% of potential interactions retaining at least one binding site. Site conservation across species was evaluated using three additional *Saccharomyces sensu strictu* species: 5,719 sites (29%) are functionally conserved in at least two of these species [31], equating to 5,503 potential transcriptional interactions retaining at least one binding site (46%). Due to the purging of detrimental mutations, we expect the effects of selection to be more apparent on sequence conservation between species than within species [32], a result that is upheld in the analyses presented below.

### Binding site conservation relates more to the importance of the regulator than the target gene

**Figure 1:**
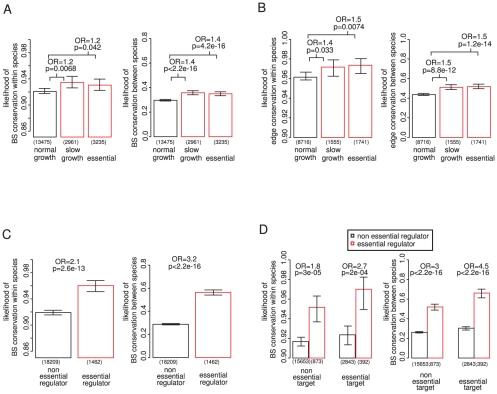
Binding site conservation relates more to the importance of the regulator than the target gene. The conservation of 19,671 experimentally determined binding sites for 119 different TFs was evaluated among 36 different strains of *S. cerevisiae* (within species conservation) and among 3 additional *sensu stricto Saccharomyces* species (between species conservation). Conservation within species implies functional conservation within all strains. Conservation between species implies functional conservation in at least 2 out of the 3 additional species. If at least one binding site for a particular transcription factor is conserved the regulatory interaction is considered as conserved. Transcription factor (TF) binding sites (A) and regulatory interactions (B) targeting genes required for growth tend to be slightly more conserved within and between species. (C) Binding sites for essential TFs are more conserved within and between species, also when controlling for essentiality of the target gene (D), this association is stronger than for the target genes (compare A and C). P-values calculated by chi square test; OR  =  odds ratio; error bars show 95% confidence intervals calculated assuming a binomial distribution and using the Wilson score interval. The number of binding sites considered is shown below each bar.

We first considered how the constraints on a binding site relate to the importance of the genes that it connects. Although the effects are quite small, both binding sites (Figure 1A) and interactions (Figure 1B) targeting genes that are required for viability or normal growth [34] are more conserved within and between species (see also Figure S1 and Figure S2). Binding sites are also more conserved in the promoters of genes that are harmful when overexpressed [35], [36] (Figure S3), consistent with the tighter regulatory control of dosage sensitive genes [37].

Similarly, the binding sites of TFs that are themselves essential for viability are more conserved within and between species (Figure 1C). This is also seen when controlling for the importance of the targeted gene (Figure 1D) or other potentially confounding factors identified below (Figure S4 and Figure S5). Moreover, binding sites of essential TFs are more conserved than the binding sites upstream of essential genes (compare Figure 1A and 1C). Hence the conservation of a binding site correlates more with the importance of the regulator than with the importance of the target gene.

### Contextual features of a promoter that predict binding site conservation

**Figure 2:**
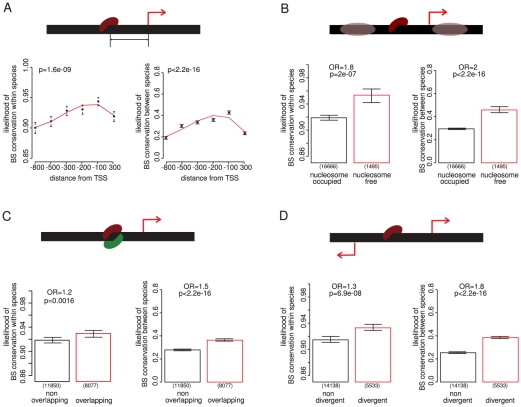
Contextual features of a promoter that predict the conservation of TF binding sites. (A) Binding sites are more conserved closer to transcription start sites – here all TF binding sites are binned into six equally populated bins sorted by distance. (B) Binding sites are more conserved if they are located in nucleosome free regions. (C) Overlapping binding sites are more conserved. (D) Binding sites are more conserved if they are located between two divergently transcribed genes. P-values were calculated using a Chi squared test, except for distance from the initiation site, where the P-value is computed using the analysis of deviance of a third degree polynomial generalized linear model fit. Bin ranges are chosen to ensure an approximately equal number of genes per bin: distance bins contain 4190, 4201, 4188, 4185, 4223, 4217 binding sites, respectively. For distance from the transcription start site, binding sites belonging to intergenic regions between two divergently transcribed genes were assigned to both promoters. Excluding these binding sites does not change the result (Figure S6A).

We next analyzed several contextual features of a binding site in a promoter to address whether they associate with site conservation. We first considered the distance to a transcription start site. Sites are more conserved closer to an initiation site, as has been previously reported for REST binding sites in human [27] (Figure 2A). The relationship is quite strong and robust to possible confounders such as gene importance and other properties of the promoter (Figure S6).

DNA is not naked in eukaryotic cells but is packaged by nucleosomes. Nucleosomes influence the accessibility of DNA and so can influence the binding of TFs. Many promoters including those of essential genes contain a DNA-encoded upstream nucleosome-free region [30], [38], and the location of binding sites in these regions is less variable between species [30]. Both within and between species comparisons show that binding sites in nucleosome-free regions are more conserved (Figure 2B). This is seen both for essential and non-essential regulators and targets (Figure S7) and supports the idea that important binding sites are often located in accessible chromatin [30], [38].

For a small number of TFs it has been reported that overlapping binding sites are more conserved [29], [39]. We confirm this observation for the complete set of yeast TF binding sites both within and between species, although the effect is quite weak (Figure 2C). Finally with respect to the promoter context of a binding site, we observe that binding sites located between two divergently transcribed genes are more conserved both within and between species (Figure 2D). These sites have the potential to influence the expression of more than one gene. The stronger conservation of binding sites in divergent promoters is not accounted for by biases in the orientation of essential genes or in the targets of essential regulators (Figure S8).

In summary, multiple aspects of promoter context associate with binding site conservation in the yeast genome, including distance to a transcription initiation site, location in a nucleosome-free region, overlap with another site, and location in a divergently transcribed promoter. Although some of these properties have been suggested from previous analyses, their generality, relative effect sizes, and independence are established here.

### Binding sites are usually less conserved when there is a potential for redundancy among sites or among different TFs

One mechanism that can reduce the importance of individual components in a biological system is genetic redundancy. For example, following the duplication of a gene, two duplicates are functionally redundant and so experience reduced selective pressure [40]. Redundancy between genes is stably maintained in genomes [41], possibly because it favors environmental or stochastic robustness. Similar redundancy may exist in transcriptional networks and influence the importance of individual binding sites and transcriptional interactions. We considered the potential for redundancy at two levels – first, among multiple binding sites for each TF, and second, among regulatory interactions mediated by different TFs. Although multiple copies of a binding site in a promoter could indicate redundancy, they may also be required for functional reasons, for example to alter the sensitivity, dynamics, or dynamic range of a transcriptional response [42]–[44]

**Figure 3:**
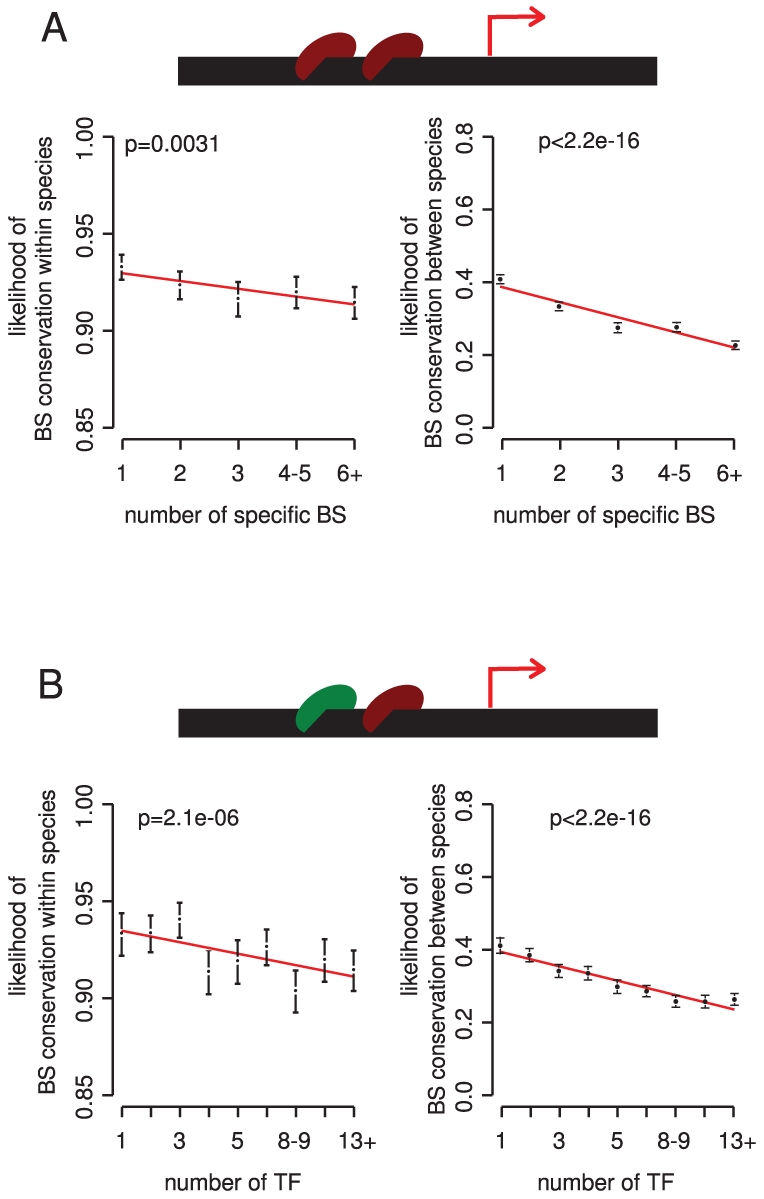
The potential for redundancy at the level of multiple binding sites for the same TF and among different TFs reduces the selective constraint on individual binding sites. Conservation within and between species is plotted against the number of binding sites for a particular TF (A) and the number of different TFs that bind to a promoter (B). Bins contain 6167, 5686, 3909, 4634 and 4808 binding sites for the 1, 2, 3, 3, 4–5, and 6+ classes, respectively in (A), and 2204, 2925, 2904, 2627, 2500, 3378, 3089, 2584, 2993 sites for the 1, 2, 3, 4, 5, 6–7, 8–9, 10–12, 13+ TF classes, respectively in (B). P-values were calculated by analysis of deviance in a generalized linear model fit.

Comparing the conservation of sites within and between species we find that more copies of a particular TF binding site in a promoter usually associate with reduced conservation (Figure 3A). This result is stronger for promoters that are only targeted by a few different TFs (Figure S9). The association is also upheld when accounting for the distance of sites to the initiation site, and for TF or target gene importance (Figure S9). Thus, in most cases the presence of multiple sites in a promoter is indeed likely to indicate (partial) redundancy among sites.

There are, however, exceptions to this trend. For several TFs, more sites in a promoter associate with stronger constraint on the individual sites (Table S2). These TFs tend to have individually weak binding sites, and binding sites present in high copy numbers (Table S3). In these cases functional regulation may require multiple copies of a binding site [42]–[44].

We next considered the potential for redundant regulation among different TFs. Consistent with a model of functional compensation among TFs, we observe that binding sites are less constrained in the promoters of genes targeted by multiple different TFs (Figure 3B). Controlling for possible confounders such as the number of different binding sites for each TF upholds this conclusion (Figure S10), and the trend is stronger when only considering essential regulators and target genes (Figure S10).

Recently, a systematic genetic interaction analysis was used to identify pairs of yeast TFs that show evidence of functional redundancy [45]. If the combined deletion of a pair of TFs causes a more severe effect on growth than expected from the individual effects of the gene deletions, then this defines a negative genetic interaction (synergistic epistasis). We reasoned that the binding sites for these pairs of TFs might also tend to be more redundant when located in the same promoter. Indeed examining binding site conservation suggests that this is indeed the case – when found in the same promoters, binding sites for TF pairs linked by a negative epistatic interaction are less conserved between species than other TF pairs (p = 9.1×10^−4^).

In summary, redundancy in transcription networks seems to exist both at the level of compensation among individual binding sites for particular TFs, and at the level of compensation among sites for different TFs. Further, TFs with partially redundant functions are more likely to have partially redundant binding sites. Similar to nodes, redundancy between edges in a network is associated with an increased robustness to perturbation.

### Low conservation of subtelomeric binding sites

**Figure 4:**
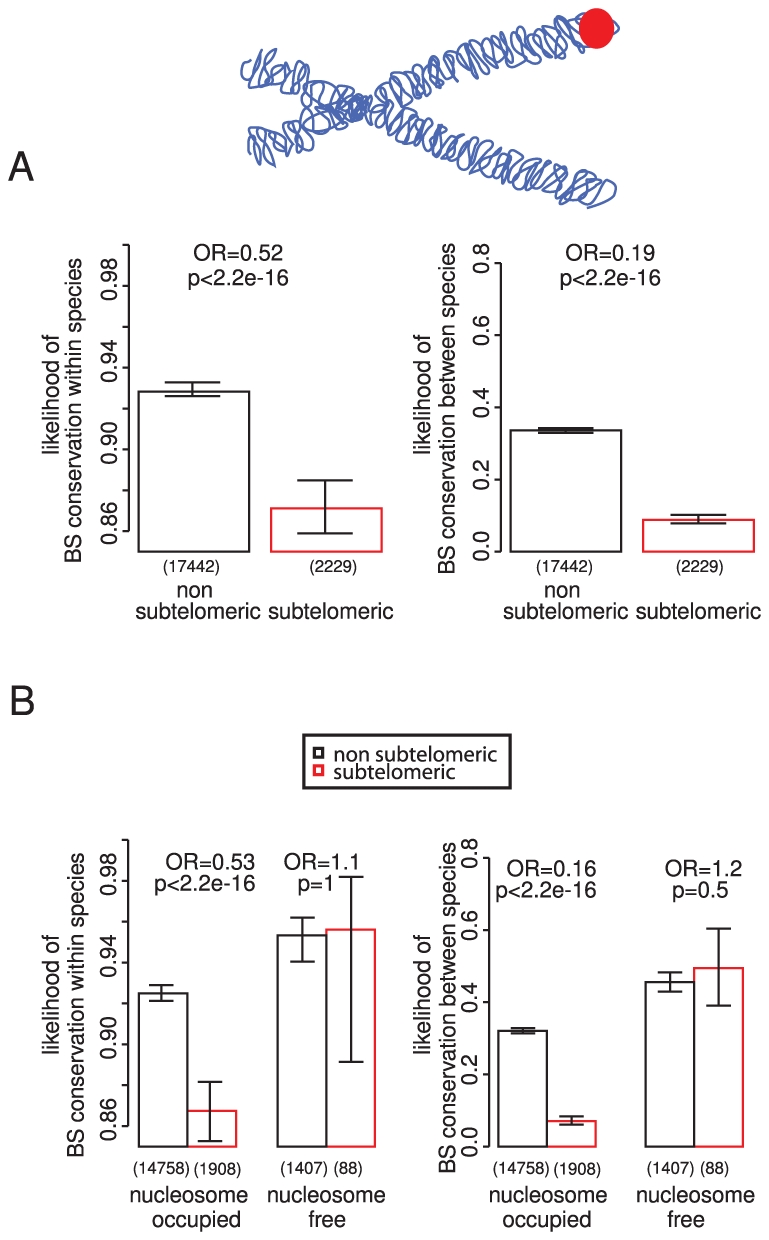
TF binding sites in subtelomeric regions and nucleosome-free regions. TF binding sites in subtelomeric regions (<40 kb from chromosome ends) are less conserved within and between species (A). However binding sites in nucleosome-free regions are similarly conserved in subtelomeric regions and in the rest of the genome (B).

Subtelomeric regions in yeast have undergone many rearrangements during evolution [46], [47] and have a higher rate of sequence divergence [48]. They are also devoid of essential genes [49] and are enriched for stress responsive loci [50], but contain many TF binding sites [51], [52]. Considering these binding sites in isolation shows that they tend to be less conserved (Figure 4A), also when controlling for possible confounders such as the number of binding sites in a promoter and gene importance (Figure S11). Also consistent with a reduced selective constraint, only 4% of subtelomeric binding sites are within nucleosome-free regions, compared to 8% of sites in the rest of the genome (p = 1.7×10^−10^). However, binding sites in subtelomeric regions that are located within nucleosome-free regions are similarly conserved to those elsewhere in the genome, showing that this subset of sites is still enriched for functionally important sites (Figure 4B).

### Network properties predict binding site conservation

**Figure 5:**
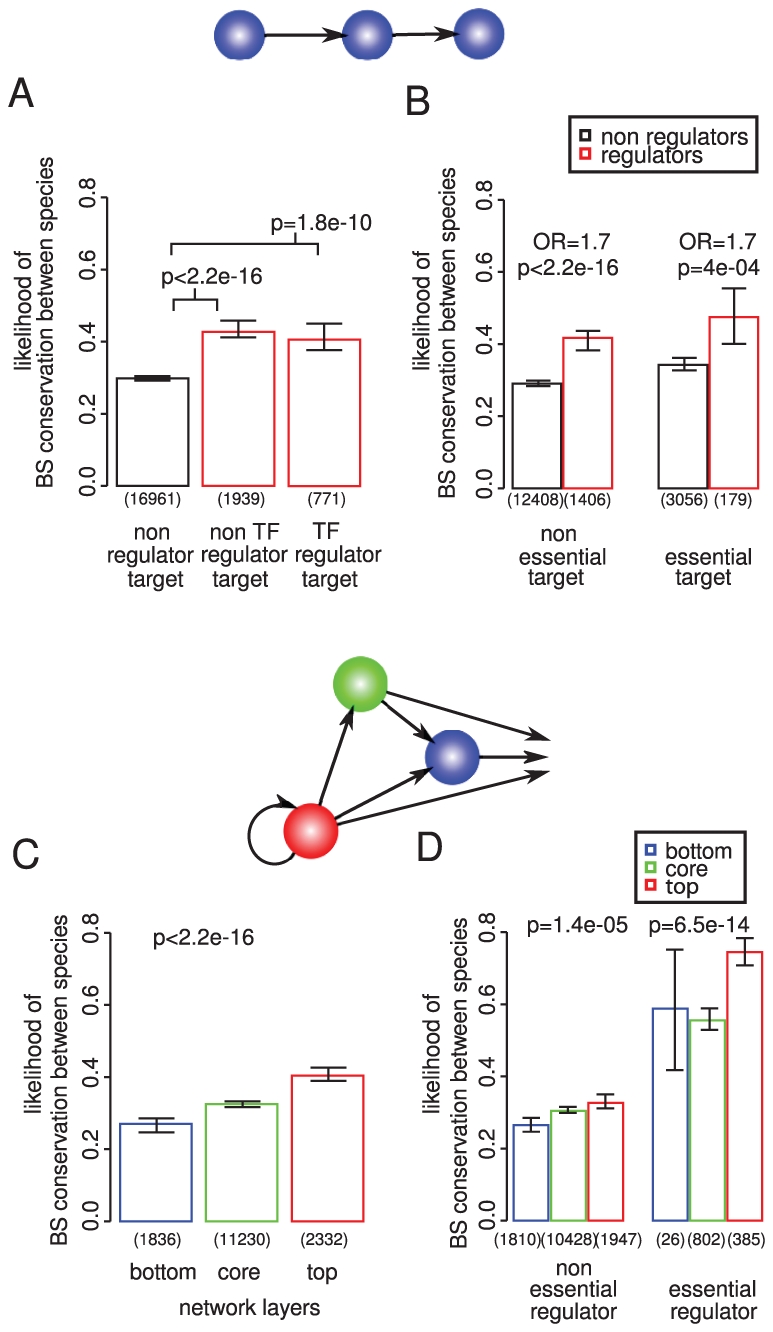
TF binding sites in the promoters of regulatory genes. TF binding sites in the promoters of regulatory genes, both TFs and non-TFs, are more conserved within and between species, consistent with the potential for a “chain effect” of mis-regulation (A). This association does not depend upon the importance of the target gene for growth (B). (C) Binding sites for TFs higher in the transcriptional hierarchy are under stronger selective constraint. This association is not due to differences in the distribution of essential TFs amongst different levels in the hierarchy (D).

The hierarchical structure of the transcriptional network of yeast [53] might also associate with differences in the importance of individual interactions. To address this we first asked whether the potential for changes in regulation to propagate in a network relates to the importance of an interaction. We compared binding site conservation in the promoters of genes that are themselves predicted to have a role in regulation [54]; a mutation that alters the regulation of a regulator has the potential to influence the expression of many downstream genes. We find that binding sites are indeed more conserved in the promoters of regulatory genes (Figure 5A). This is true both for TF regulators and non-TF regulators such as signaling proteins [54] (Figure 5A), and is upheld when accounting for other known influences (Figure S12), for example it is not dependent on the essentiality of the target gene (Figure 5B).

We next compared the conservation of binding sites and edges for TFs classified in the top, core, and bottom layer of the transcription hierarchy [53]. We find that binding sites for TFs in the top of the hierarchy are more conserved (Figure 5C). The association with hierarchy is stronger for more important targets and regulators, but also observed for non-essential regulators (Figure 5C) and targets (Figure S13), and for interactions that target both regulators and non-regulators in the network (Figure S13). Interactions mediated by TFs at the top of a regulatory hierarchy tend, therefore, to be under stronger constraint in yeast.

### Stronger binding sites are more conserved

More important regulatory interactions may in general have evolved to use stronger binding sites across a genome. This would allow more robust discrimination of these sites from the genomic background, and predicts that changes in stronger sites should in general be more detrimental. There is some evidence for this based on an analysis of the conservation of a limited number of binding sites in *Drosophila* species [28]. Further, in yeast it has been previously noted that the promoters of essential genes and divergent promoters tend to have fewer binding sites [26], and that promoters with fewer sites tend also to have stronger sites [26], which is also consistent with this hypothesis.

**Figure 6:**
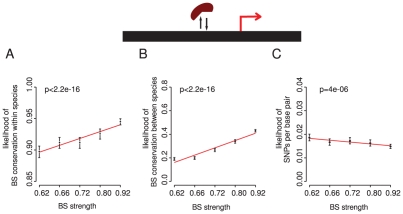
Stronger binding sites more closely matching the intrinsic binding preference for a TF are more conserved within and between species. The proportion of conserved sites is shown for six equally populated bins of sites within (A) and between (B) species. The number of sequence changes per base pair within species is also shown (C).

Using the complete set of TF binding sites in yeast there is indeed a strong relationship between the strength of a site (its optimality) and site conservation (Figure 6A, 6B). Binding sites that more closely match the intrinsic binding preference for a TF are more conserved within and between species. This is also observed when considering the number of sequence changes without taking into account their effect on a binding site score (Figure 6C and Table S2), indicating that the association is not dependent upon our definition of functional conservation. We conclude that stronger binding sites are more conserved within and between species in yeast.

### Similar trends are seen when considering the number of sequence changes per base pair and TFs with *in vitro* validated binding preferences

To account for possible biases that might derive from the *in vivo*-defined TF binding preference models used in our analyses [31], we also considered binding site conservation in terms of the number of sequence changes per base pair in each site. Repeating all of our analyses using this alternative definition of site conservation gives very good agreement with the results reported here (Table S1), showing that our conclusions do not depend on the use of a hard threshold for the functional conservation of a binding site.

A further possible confounder could be regional variation in sequence divergence across different promoters due to either mutation rate variation or additional selection biases [55]. To address this, we compared the sequence conservation bases within binding sites to that of the bases immediately flanking each site. This analysis confirmed that the features reported here are associated with variation in the conservation of the binding sites themselves (Figure S14 and Figure S15).

As an additional control, we also repeated all of the analyses on a subset of TFs where the *in vivo* defined binding site preferences have been confirmed by *in vitro* binding specificity analysis [56]. Analyzing the conservation of the genomic binding sites for these TFs confirms our findings, both when using a threshold to define binding site conservation and when analyzing the number of sequence changes per base pair within binding sites for these TFs (Table S1). Our results are thus robust to possible biases in the complete set of binding site preference models.

### Similar properties apply to the interactions of most TFs

To investigate how general the associations reported here are for different TFs, we also analyzed the binding sites of each TF individually (Table S2). For nearly all features, the global relationship is also upheld for a majority of TFs when they are analyzed individually. One notable exception, as described above, is the number of binding sites per TF, where for particular TFs more instances of a binding site are associated with stronger site conservation rather than reduced conservation as expected due to redundancy. However, in general we conclude that the features described here as associated with stronger evolutionary constraints on transcription interactions apply similarly to binding sites for most TFs in the genome.

### An integrated model predicts binding site conservation across a genome

Given the generality of our findings, we asked whether we could use the identified features to predict the conservation of binding sites for all TFs in all promoters of the genome. For this purpose we used a generalized linear modeling (GLM), because it can accommodated the binary response variable of sequence conservation, and allows both linear and non-linear effects to be estimated for both continuous and categorical explanatory variables. To assess the predictive power of each feature alone and in combination we used a receiver operating characteristic (ROC) curve analysis, with ten-fold cross-validation.

**Figure 7:**
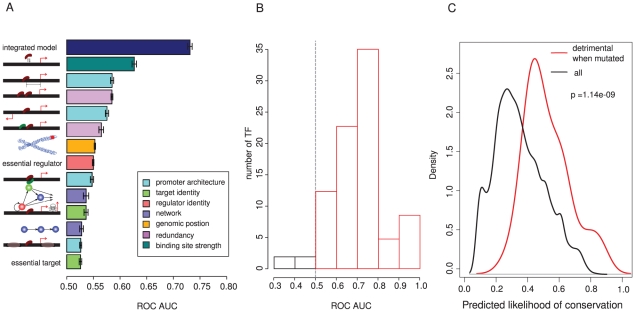
An integrated model provides good predictive performance of binding site conservation between species. (A) Predictive performance of individual features associated with binding site conservation, and of the final integrated model evaluated using ten-fold cross-validation and quantified using the area under the curve (AUC) of a receiver operating characteristic (ROC) plot. Mean and standard error of the AUC is shown for each feature. (B) The distribution of AUC values for the integrated model evaluated individually for each of 119 TFs. This shows that the same features provide good predictive performance for the conservation of binding sites for most TFs in a genome. (C) Comparison of the distribution of integrated model prediction scores for a subset of 44 binding sites where mutation of the site has been determined experimentally to be detrimental (see Materials and Methods) to the overall distribution of sites. Density is the probability density function of each distribution estimated using the 'density' function of R. The integrated model was constructed using a generalized linear model as described in the Materials and Methods.

We first assessed the predictive performance of each feature alone, considering predictions both between (Figure 7) and within (Figure S16) species. As expected due to the lower number of sequence changes within a species and the purging of deleterious mutations between species, the predictions are better for the between species data. However, in both cases the qualitative results are very similar, with binding site strength the best single predictor of conservation. After strength, features related to redundancy, promoter architecture, and position are the next most predictive, followed by the importance of the regulator, network properties, and the importance of the target gene.

Combining information from multiple features substantially improves the overall predictive performance (Figure 7, Figure S16, Figure S17, Figure S18). We used a stepwise strategy to construct a predictive model, starting from a model involving all terms and their first order interactions and then removing terms that gave no significant improvement in the model. The final model includes the following terms, listed in the order of their effect on deviance when they are individually excluded (Table S4 and Table S5): binding site strength, location in a subtelomeric region, importance of the regulator, divergent promoters, distance from a start site, identity of the target gene as a regulator, overlapping binding sites, hierarchy of the regulator, and the number of transcription factors targeting a promoter. No interaction terms were found to significantly contribute to the model. In a ten-fold cross-validation analysis, the conservation of binding sites between species was predicted with an area under the ROC curve (AUC) of 0.73 +/− 0.01 (Figure 7A). This means that for a randomly chosen combination of a conserved and a non-conserved binding site, there is a 73% chance that the model will correctly classify them.

Strikingly, this quite simple model predicts conservation similarly well for nearly all of the different yeast TFs (Figure 7B). This suggests that similar principles predict binding site importance for many different TFs in a genome.

### Experimentally defined detrimental binding site mutations verify the model

Finally, to provide an independent assessment of the model, we performed an extensive literature curation to identify binding sites in the dataset that have been evaluated as functionally important in laboratory experiments. In total we identified 44 binding sites where mutations in the site have been found to alter the expression of a neighboring gene, or to cause a fitness defect such as a cell cycle or growth defect (these sites are listed as a resource in Table S6). The distribution of integrated model scores for these binding sites is strongly shifted to high values (Figure 7C). This shows that the integrated model predicts deleterious binding site losses that have been identified by sequence conservation and those identified by direct experimental perturbation.

## Discussion

Biological systems are defined by their components, but also by the interactions among these components. Likewise, mutations can affect the components, but also their interactions, and an important challenge is to understand when mutations that alter interactions are most likely to be detrimental [11], [12]. In this study we have used the transcriptional network of yeast as a model system to address this question, and an integrative analysis to identify the properties that define the most conserved transcription interactions in a genome.

Within a genome, each transcription factor associates with a very large number of sites [15]–[21]. This is not surprising given the short and degenerate sequences that they recognize and the large size of eukaryotic genomes [14]. What distinguishes the binding sites that are most important for the fitness of an organism? Based on the analysis here we can offer the following principles for these sites in yeast (Figure 7A). First, and most strikingly, stronger binding sites are more important for the fitness of an organism. Second, important binding sites tend to be located closer to a transcription start site. Third, for many (but not all) TFs the presence of multiple copies of a binding site in a promoter reduces the constraint on the individual sites. Fourth, sites are more conserved in divergent promoters, in nucleosome free regions and when overlapping. Fifth, bindings sites are less conserved if there is a potential for redundant regulation by additional TFs. Sixth, binding sites are less conserved in subtelomeric regions. Seventh, the binding sites of essential TFs are more conserved, and to a lesser extent so are binding sites in the promoters of essential genes. Eighth, binding sites are more conserved if they are located in the promoters of regulatory genes, and for TFs at the top of a regulatory hierarchy.

Our analysis shows therefore that there are common properties associated with many of the most important transcription interactions in a genome. The association between site strength and importance is particularly interesting, as it suggests that evolution has favored stronger binding sites for the most important interactions. This likely facilitates their discrimination from the genomic background. Stronger sites are also less likely to evolve *de novo* in a genome, so compensation (‘turnover’ or ‘network-level conservation’ [28], [57]–[59]) may be less likely for these sites. However, the tendency to gain new binding sites does not account for the relationship between site strength and conservation (Figure S19).

By combining features we constructed a model that predicts binding site conservation with quite good performance across all promoters in the yeast genome. This single model predicts conservation similarly well for many different TFs, and also recovers binding sites that have been experimentally validated as functionally important. Thus, despite the potential for regulatory diversity and complexity there are actually common properties that can be used to predict many of the most important transcription interactions in a cell.

## Materials and Methods

### Transcription regulatory network

Transcription factor binding sites analyzed in this manuscript derive from a comprehensive chromatin immunoprecipitation study using 203 TFs [16], with binding site locations and motifs taken from [31]. We used a binding confidence cut-off of p = 0.005 and no conservation constraints across species in the definition of physical binding sites. Gene start sites were determined using data from [60] when available. Each binding site was assigned to the nearest downstream gene (within 1000 bp), and to both genes in the case of divergent promoters.

### Natural variation within transcription factor binding sites

To identify sequence polymorphisms (SNPs) within TF binding sites we used the genome sequences of 36 wild and domestic *S. cerevisiae* strains [33]. The transcriptional regulatory map coordinates were updated using the October 10th 2007 release of the *Saccharomyces* Genome to match those used by Liti et al. Only SNPs with a high sequence quality confidence level (p<1×10^−3^) were considered for the analysis. Insertions and deletions were not considered because we find them to be unreliable in this dataset (our unpublished analysis). A total of 2,182 binding sites (11.1%) contained at least one SNP in at least one strain.

### Within species binding site conservation

Following [16], a binding site was considered as functionally conserved if it scores at least 60% of the maximum possible score of its position specific scoring matrix (PSSM) model, with the score defined as:
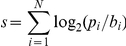
where p_i_  =  likelihood of base at position i according to the PSSM; b_i_  =  background frequency of base i; N  =  number of base pairs in the motif. According to this criterion a total of 18090 (92%) sites are functionally conserved in all strains.

### Between species binding site conservation

Between species binding site conservation was evaluated as in [31], requiring functional conservation in at least 2 of 3 additional *sensu strictu Saccharomyces* species. According to this criterion a total of 5719 binding sites (29%) are functionally conserved.

### Transcriptional interaction (“edge”) conservation

A regulatory interaction between a transcription factor and its target gene (transcription network edge) is considered as conserved if at least one of the binding sites for the transcription factor is functionally conserved in the promoter region of the target gene.

### Gene importance

Essential genes and genes required for normal growth were taken from [34]. Genes harmful when overexpressed were defined in two studies [35], [36] and compiled in [61].

### Nucleosome occupancy

Promoters with and without nucleosome free regions were retrieved from [30], considering 150 bp before the start site.

### Subtelomeric regions

Genome regions within 40 kb of the chromosome ends where considered as subtelomeric [48].

### Regulators

Genes with regulatory activity (transcription factors and signaling genes) were taken from [54].

### Transcription hierarchy

TFs were classified into three hierarchical levels according to the analysis of [53]. We excluded from the classification regulators that were not uniquely assigned to one of these three levels.

### Binding site strength

We used the PSSM score of each binding site instance normalized to maximum possible score of the PSSM as a measure of its strength (or optimality).

### Number of sequence changes per base pair

For this analysis the fraction of single nucleotide polymorphisms over the number of base pairs in each binding site is considered. Bases located in gaps of the motif are excluded from the analysis. Overlapping binding sites were excluded from this analysis.

### *In vitro* confirmed binding site motifs

PSSM models from [31] that show high similarity (Pearson's correlation coefficient >0.7) with PSSMs defined by an *in vitro* protein binding microarray experiment [56] were considered as a separate higher-confidence subset of PSSMs.

### Individual transcription factor analysis

The relationship between binding site importance and each determinant was also assessed on a per TF basis. For distance from the transcription start site, TFs with at least four instances at different distances were selected for the analysis. For the other discrete variables at least two values are required for selection. For categorical variables at least one instance for each category is required for TF selection.

### Integrative model

Binding site conservation within and between species was predicted using a generalized linear model (GLM). This statistical model was chosen because it can properly account for the binary response variable and allows the estimation of both linear and non-linear effects for continuous and categorical predictor variables at the same time. The GLM specifies the relationships between a linear predictor (η) and a set of the explanatory variables (*x_i_*) by estimating the coefficients β*_i_* from the data:
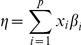



The linear predictor η is not directly related to the predicted response µ, the likelihood of binding site conservation. Instead, the response is related to the linear predictor through a link function, 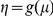. The canonical link function in the case of a binary response is the logit function:
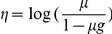



We used the ‘glm’ function in R with the option ‘family  =  binomial’. This function calculates maximum likelihood estimates of the parameters using a iteratively re-weighted least square algorithm. Distance from the transcription start sites was modeled with 3 parameters specifying a third degree polynomial curve using the ‘poly’ function in R. Hierarchy of the regulator was considered as an ordered categorical variable with network layers ordered as ‘top’ > ‘core’ > ‘bottom’ and modeled using 2 parameters estimating both linear and quadratic trends.

The final model was selected with a stepwise strategy, starting with all the feature terms and their first order interaction terms. At each step the terms that did not significantly improve model performance were dropped one by one starting from the least significant interaction term. Analysis of deviance was used to compare the simplified model to the previous one and a chi-square test (with a p<0.05 threshold) used to evaluate the significance of the drop in the model performance.

The model was used as a classifier to predict binding site conservation in a ten-fold cross-validation analysis, i.e. the model was repeatedly fitted to a subset of the data (training set) and used to predict the other subset (test set). The area under the Receiver Operating Characteristic curve (ROC AUC) was used to assess predictive performance.

### Literature curation of binding sites experimentally validated as influencing gene expression or fitness

To identify binding sites where loss of the site has been experimentally demonstrated to have an influence on gene expression or fitness we started from the *S. cerevisiae* Promoter database (SCPD) compilation of binding sites studied in small-scale studies [62]. For each binding site in this database we evaluated from the original publications whether the binding site has been mutated or deleted in its normal promoter context, and whether this inactivation has been demonstrated to have an effect on gene expression or fitness (e.g. a growth or cell cycle defect). In total, 44 binding sites from the MacIsaac dataset were identified that fulfilled these criteria, from a search through >150 publications. These sites are listed in Table S6.

### Transcription factors with negative genetic interactions

Transcription factors with negative genetic interactions were identified as those with an E-MAP score <−3 [45].

### Statistical analysis

All statistical analyses were performed in R (http://www.r-project.org/). The Chi Square test or Fisher's exact test was used to test for independence with categorical data, and a binomial generalized linear model was used to test trend significance for discrete variables. Empirical p-values calculated using label shuffling gave very similar results (not shown).

## Supporting Information

Figure S1 Binding sites are more conserved in the promoters of essential genes when controlling for possible confounders. The plots show the fraction of conserved binding sites within and between species (as defined in Materials and Methods) and the number of single nucleotide polymorphisms (SNPs) per base pair in binding sites within species. The higher conservation of binding sites and edges targeting essential genes is stronger for divergent promoters but also observed for non non-divergent promoters (A). The association is upheld for both essential and non-essential TFs (B), and for nucleosome free regions (C). The association is stronger for TFs higher in the regulatory hierarchy (D) and it is upheld when controlling for potential redundancy among binding sites (E) and transcription factors (F), and for distance from the transcription start site (G).
(PDF)

Figure S2 Regulatory interactions targeting essential genes are more conserved when controlling for possible confounding factors. The fraction of conserved edges (as defined in Materials and Methods) both within and between species are shown, when controlling for regulation by an essential regulator (A), or when the regulatory edge is constituted by a non redundant binding site (B). The association is stronger for TFs higher in the hierarchy (C) and it is upheld controlling for the number of transcription factors regulating a promoter (D).
(PDF)

Figure S3 Binding sites and regulatory edges are more constrained if they target genes that are harmful when overexpressed. The plots show the fraction of conserved binding sites (A) and conserved edges (B) within and between species (as defined in Materials and Methods).
(PDF)

Figure S4 The binding sites of essential regulators are more conserved when controlling for possible confounders. The plots show the fraction of conserved binding sites both between and within species and the number of single nucleotide polymorphisms (SNPs) per base pair in binding sites within species. The increased constraint on the binding sites for essential TFs is more apparent for non-divergent promoters but it is also significant for divergent promoters (A). The association is also upheld for both essential and non-essential target gene promoters (B), when controlling for nucleosome occupancy (C) and for position in the network hierarchy (D). The association is strong for non-redundant binding sites (E) and when there is no possibility of redundancy among transcription factors (F), but is is still upheld in potential cases of redundancy, and when controlling for distance from the transcription start site (G).
(PDF)

Figure S5 Essential regulator edges are more conserved controlling for potential confounders. The regulatory interactions of essential regulators are more conserved whether they target essential genes or non essential ones both within and between species (A). The association is stronger for TFs higher in the hierarchy but it is also present in lower layers (B). The association is also stronger for edges with non-redundant binding sites but it is also present for edges with redundant binding sites (C), is stronger when only one transcription factor regulates a promoter (D).
(PDF)

Figure S6 Binding sites closer to a transcription start site are under stronger selective constraint when controlling for potential confounders, including divergent promoters (A), essentiality of the regulator (B), and of the target gene (C).
(PDF)

Figure S7 Binding sites in nucleosome free regions are more conserved also controlling for essentiality of the target gene.
(PDF)

Figure S8 Binding sites in divergent promoters are more conserved even when accounting for target gene (A) and regulator essentiality (B).
(PDF)

Figure S9 The potential for redundancy among binding sites relaxes constraints on individual binding sites. The plots show the fraction of conserved binding sites both between and within species (as defined in Materials and Methods) and the number of single nucleotide polymorphisms (SNPs) per base pair in binding sites within species. The effect of binding site redundancy is higher at a lower total number of binding sites in the promoters (A). Binding site redundancy relaxes constraint on binding sites at different distances from the transcription start site (B). The association between redundancy and reduced constraint is upheld when considering essential regulators (C) and essential target genes (D).
(PDF)

Figure S10 The potential for redundancy among transcription factors relaxes evolutionary constraints on TF binding sites. The plots show the fraction of conserved binding sites both between and within species (as defined in Materials and Methods) and the number of single nucleotide polymorphisms (SNPs) per base pair in binding sites within species. The association with transcription factor redundancy is stronger for non-redundant binding sites but it is also present for potentially redundant ones (A). Controlling for distance from the start sites (B), essentiality of the regulator (C) and essentiality of the target gene (D) also upholds the result.
(PDF)

Figure S11 The reduced conservation of binding sites in sub-telomeric regions. The plots show the fraction of conserved binding sites both between and within species (as defined in Materials and Methods) and the number of single nucleotide polymorphisms (SNPs) per base pair in binding sites within species. The reduced conservation of binding sites in sub-telomeric regions is upheld when controlling for confounders such as binding site redundancy (A), divergent promoters (B), regulator (C), and target essentiality (D).
(PDF)

Figure S12 The increased constraint on binding sites in the promoters of regulatory genes is upheld when controlling to possible confounders such as the essentiality of the regulator (A), and redundancy among binding sites (B). The association is stronger when more than one TF targets the promoter (C), and upheld when controlling for the distance to a start site (D).
(PDF)

Figure S13 Binding sites for TFs higher in the regulatory hierarchy are more constrained when controlling for possible confounders such as identity of a target as a regulator (A), target importance (B), regulator importance (C), binding site redundancy (D), and the number of TFs that target a promoter (E). Indeed the association is stronger when controlling for target or regulator importance and for non-redundant binding sites.
(PDF)

Figure S14 Nucleotide conservation compared inside binding sites to that in the 10 nucleotides downstream of each site (excluding nucleotides located within a known binding site).
(PDF)

Figure S15 Comparison of the number of sequence changes per base pair within binding sites (BS) to that in the 10 bp downstream of each site (excluding bases located within known binding sites) for various properties.
(PDF)

Figure S16 Predicting binding site conservation within species gives a similar qualitative relative performance as predicting conservation between species (see Figure 7), although the predictive power, as expected, is generally lower. The model shown was trained on within species conservation data and used to predict within species conservation (see Materials and Methods for further details). The predictive power is measured by the area under a receiver operating characteristic curve (ROC AUC, see Materials and Methods).
(PDF)

Figure S17 Precision-Recall plot showing the performance of the integrated model in predicting binding site conservation between species.
(PDF)

Figure S18 Predictive performance of the model for predicting the between species conservation of binding sites, evaluated after the addition of each additional variable. The predictive power is measured by the area under a receiver operating characteristic curve (ROC AUC). The mean and standard error of the AUC is shown for each model of increasing complexity.
(PDF)

Figure S19 Stronger binding sites are more conserved when controlling for the gain of new binding sites (turnover). Stronger binding sites are less likely to arise *de novo* from the genomic background with the result that their stronger conservation could partially reflect a lower probability of compensation. To test this we scanned the promoters of the different *S. cerevisiae* strains and annotated when a new instance of a TF appeared in a promoter. We then analyzed the relationship between BS strength and BS conservation within species in the presence or absence of an alternative (gained) BS in the promoter of at least one of the strains. This shows that stronger binding sites are more conserved, even when taking into account the potential for compensation.
(PDF)

Table S1 Considering a subset of 24 TFs with *in vitro* confirmed binding site preferences supports the reported associations. Effects and significance are quantified using a generalized linear model (see Materials and Methods for further details). For categorical variables effect is the change in log odds of binding site conservation or base pair changes between the two categories, while for linear fits of discrete variables the effect is the change in log odds of conservation per unit of the variable. In this case distance from the transcription start site has been modeled with a linear fit up to the transcription start site instead of an orthogonal polynomial fit to simplify the comparison of the coefficients among the different datasets. Note that in the SNPs per base pair analysis the sign of the effect is the opposite to the within species and between species conservation because higher number of SNPs equates to a lower conservation. BS – binding site.
(DOC)

Table S2 Most of the associations reported in this manuscript are also observed for a majority of individual TFs when examined in isolation. The proportion of individual TFs showing the reported effect are shown, also when restricting to TFs that show an effect significant at the 5% level. TSS – transcription start site.
(DOC)

Table S3 Transcription factors whose binding sites are more conserved when present in multiple copies in a promoter have weaker binding sites and more binding sites in each promoter. Wilcoxon test p-values on testing the hypothesis that the selected transcription factors have lower binding site strength and higher number of binding sites compared to transcription factors are shown for all transcription factors that show a significant (p<0.05) positive relationship between conservation and the number of binding sites in the promoter are selected.
(DOC)

Table S4 Summary of the fit of the integrated model for binding site conservation between species. The model was fitted using a generalized linear model framework (see Materials and Methods for further details). For distance from TSS a third degree orthogonal polynomial fit was used (see method). Hierarchy of the regulator was modeled as an ordered factor with two levels and orthogonal polynomial contrasts have been fitted. The estimated effects for categorical variables represent the log odds of the conservation between two categories, while for linear fit of discrete variables they represent the log odds per unit of variable. The table also shows coefficient, standard errors, the z-values and the p-values. The BS strength (score) is the most important determinant of binding site conservation but the other determinants still independently explain part of binding site conservation.
(DOC)

Table S5 Comparison between the final model and simpler models that exclude each of the analysed features. The analysis of deviance tables show degrees of freedom, residual unexplained deviance, the likelihood ratio test and its p-value. Binding site strength is the feature that causes the highest decrease in explanatory power (increase in residual deviance) when excluded from the model. Each of the features contributes significantly to the explanatory power of the model.
(DOC)

Table S6 Binding sites experimentally validated as important for gene expression or fitness. The list includes binding sites from the systematic dataset supported by an independent experimental report. Each BS has been mutated or deleted and a deleterious effect on the expression of the neighboring gene or fitness verified.
(DOC)
