# Supplementary figures and images for: Integrated Genome-Scale Prediction of Detrimental Mutations in Transcription Networks
Source: PLoS Genet. 2011 May 26;7(5):e1002077. doi: 10.1371/journal.pgen.1002077 (PMC3102745; doi:10.1371/journal.pgen.1002077)

A

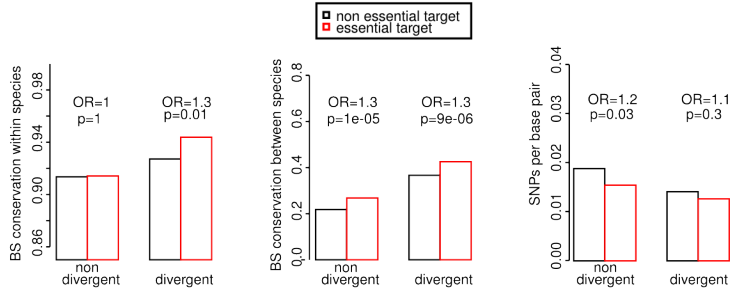

B

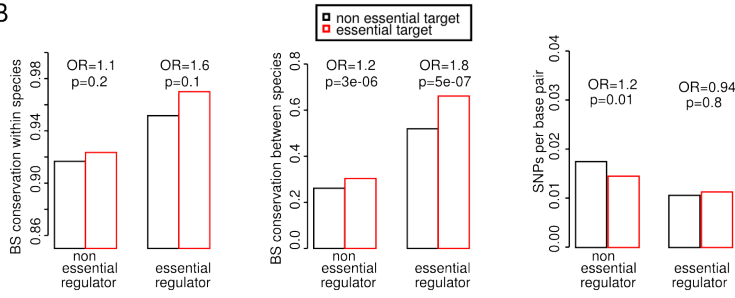

C

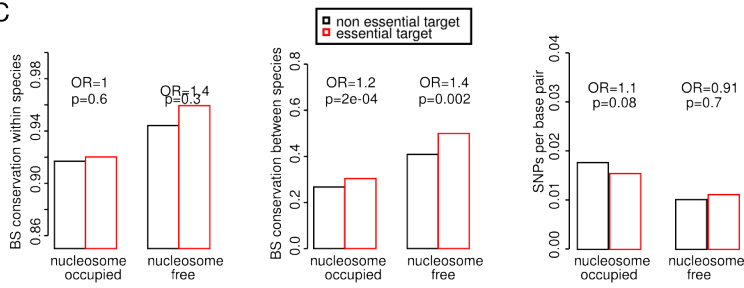

D

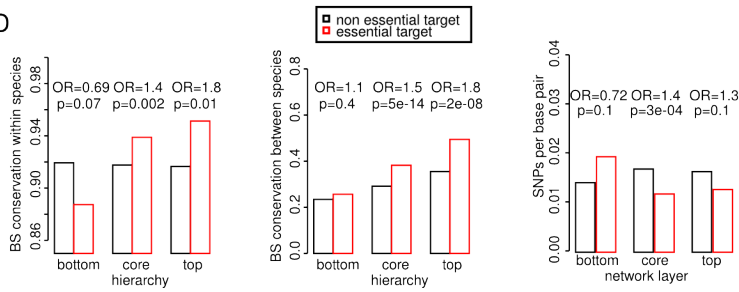

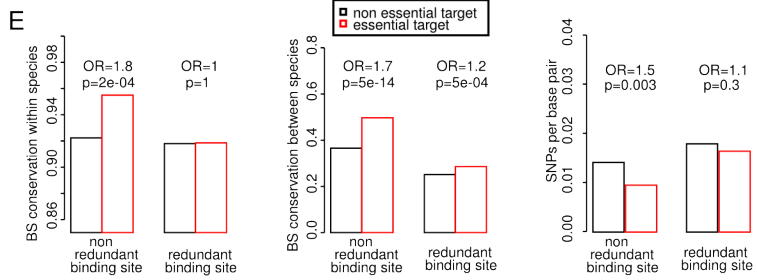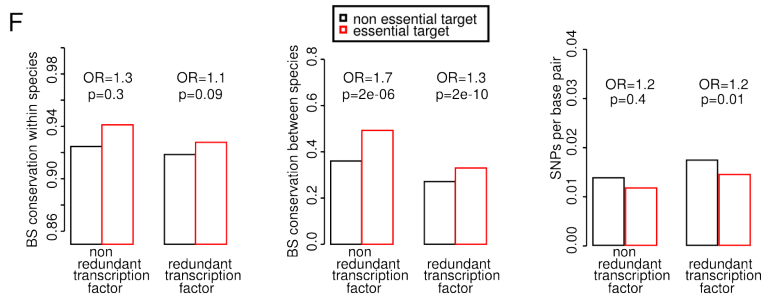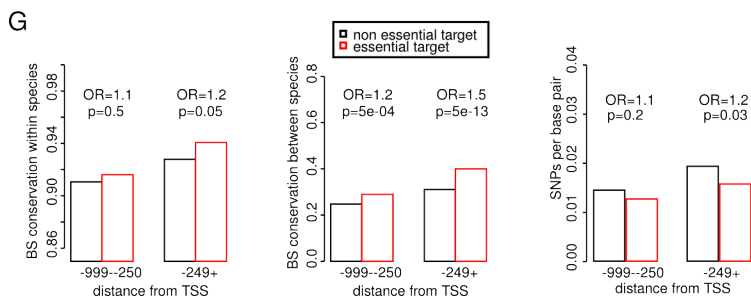

Supplement: Figure S1 — Binding sites are more conserved in the promoters of essential genes when controlling for possible confounders. The plots show the fraction of conserved binding sites within and between species (as defined in Materials and Methods) and the number of single nucleotide polymorphisms (SNPs) per base pair in binding sites within species. The higher conservation of binding sites and edges targeting essential genes is stronger for divergent promoters but also observed for non non-divergent promoters (A). The association is upheld for both essential and non-essential TFs (B), and for nucleosome free regions (C). The association is stronger for TFs higher in the regulatory hierarchy (D) and it is upheld when controlling for potential redundancy among binding sites (E) and transcription factors (F), and for distance from the transcription start site (G). (PDF) [file pgen.1002077.s001.pdf]

A

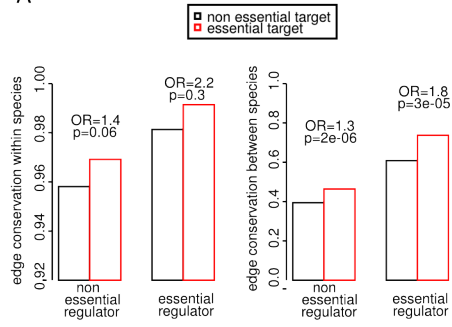

B

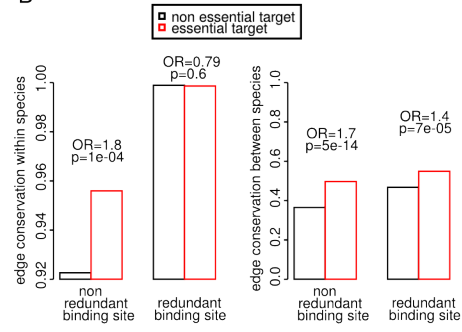

E

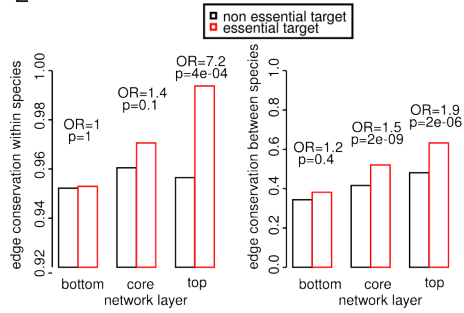

G

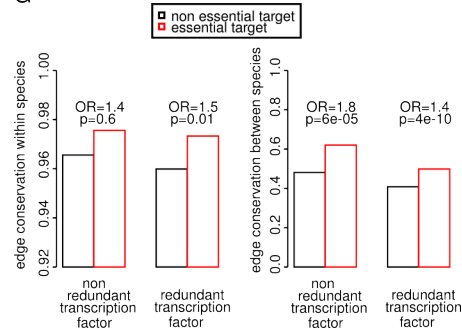

Supplement: Figure S2 — Regulatory interactions targeting essential genes are more conserved when controlling for possible confounding factors. The fraction of conserved edges (as defined in Materials and Methods) both within and between species are shown, when controlling for regulation by an essential regulator (A), or when the regulatory edge is constituted by a non redundant binding site (B). The association is stronger for TFs higher in the hierarchy (C) and it is upheld controlling for the number of transcription factors regulating a promoter (D). (PDF) [file pgen.1002077.s002.pdf]

A

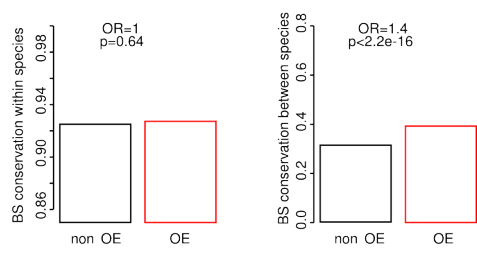

B

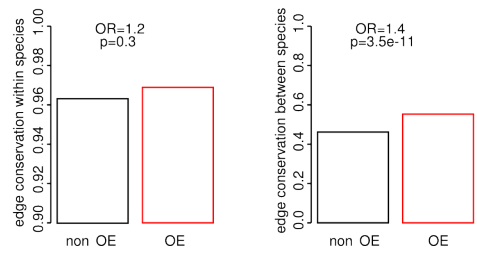

Supplement: Figure S3 — Binding sites and regulatory edges are more constrained if they target genes that are harmful when overexpressed. The plots show the fraction of conserved binding sites (A) and conserved edges (B) within and between species (as defined in Materials and Methods). (PDF) [file pgen.1002077.s003.pdf]

A

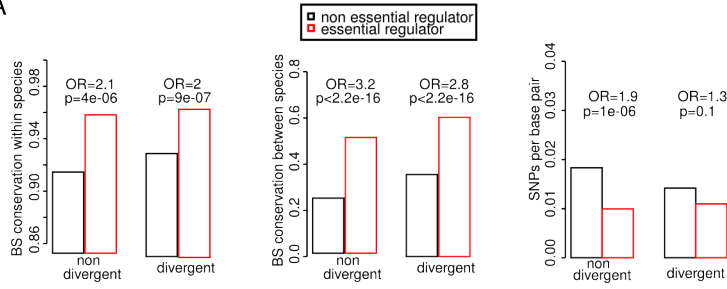

B

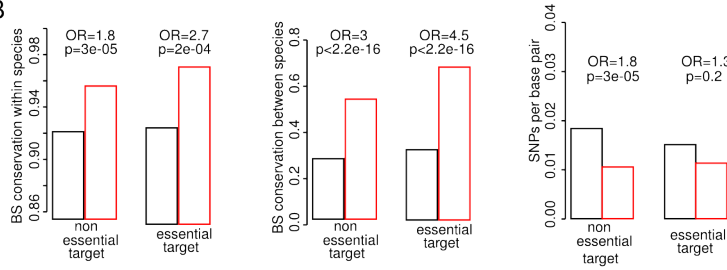

C

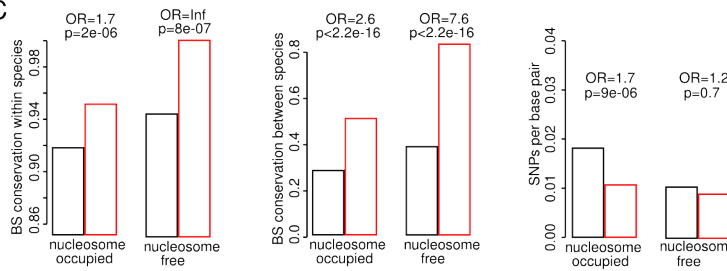

D

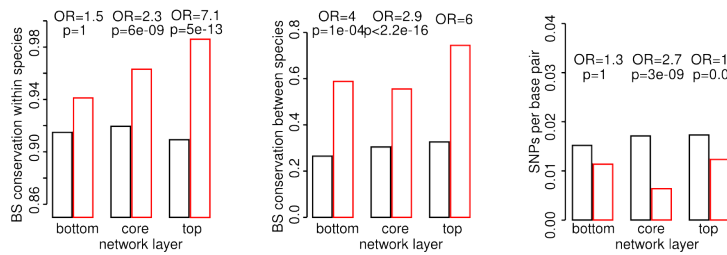

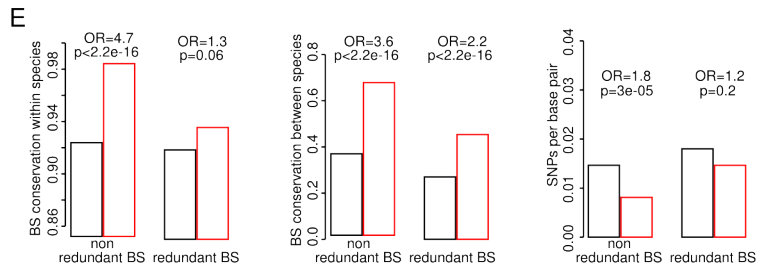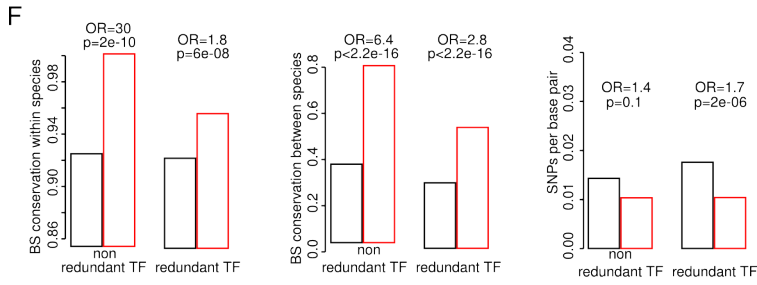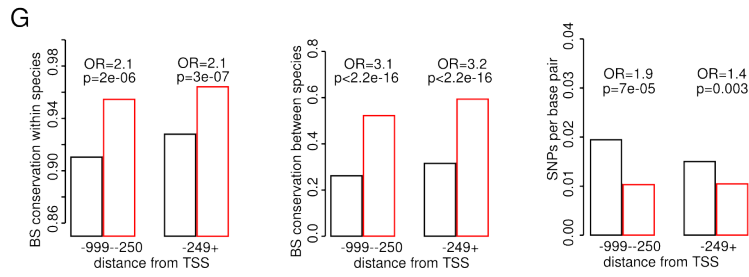

Supplement: Figure S4 — The binding sites of essential regulators are more conserved when controlling for possible confounders. The plots show the fraction of conserved binding sites both between and within species and the number of single nucleotide polymorphisms (SNPs) per base pair in binding sites within species. The increased constraint on the binding sites for essential TFs is more apparent for non-divergent promoters but it is also significant for divergent promoters (A). The association is also upheld for both essential and non-essential target gene promoters (B), when controlling for nucleosome occupancy (C) and for position in the network hierarchy (D). The association is strong for non-redundant binding sites (E) and when there is no possibility of redundancy among transcription factors (F), but is is still upheld in potential cases of redundancy, and when controlling for distance from the transcription start site (G). (PDF) [file pgen.1002077.s004.pdf]

A

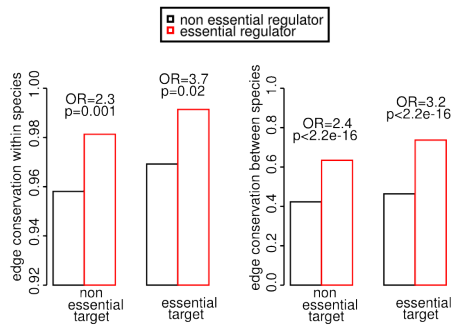

B

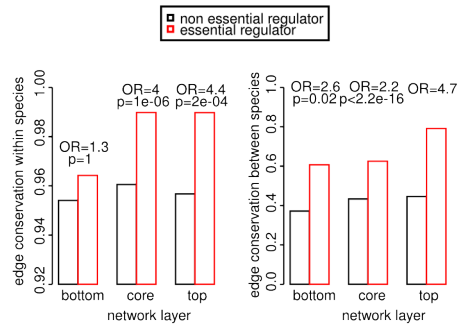

C

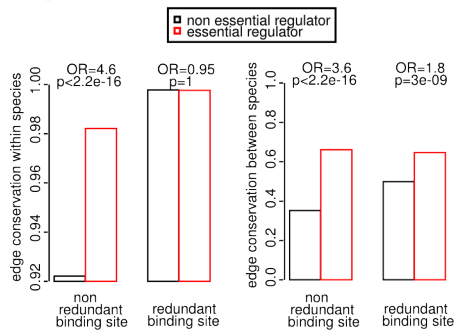

D

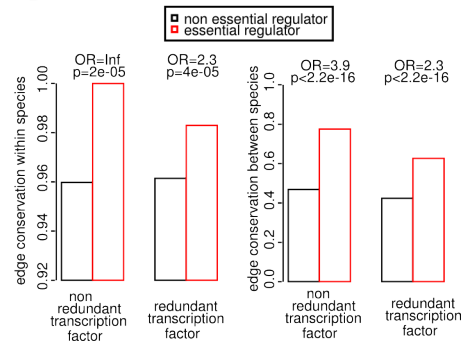

Supplement: Figure S5 — Essential regulator edges are more conserved controlling for potential confounders. The regulatory interactions of essential regulators are more conserved whether they target essential genes or non essential ones both within and between species (A). The association is stronger for TFs higher in the hierarchy but it is also present in lower layers (B). The association is also stronger for edges with non-redundant binding sites but it is also present for edges with redundant binding sites (C), is stronger when only one transcription factor regulates a promoter (D). (PDF) [file pgen.1002077.s005.pdf]

A

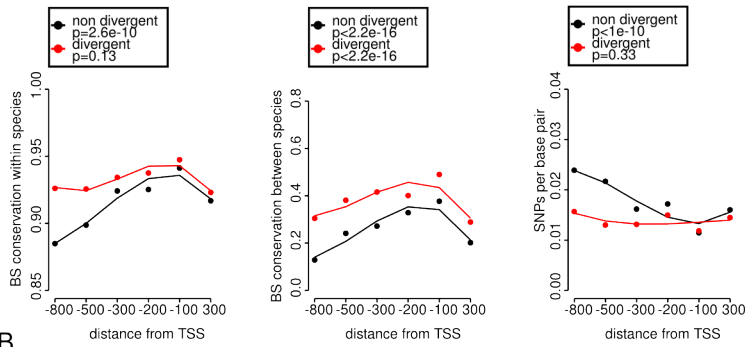

B

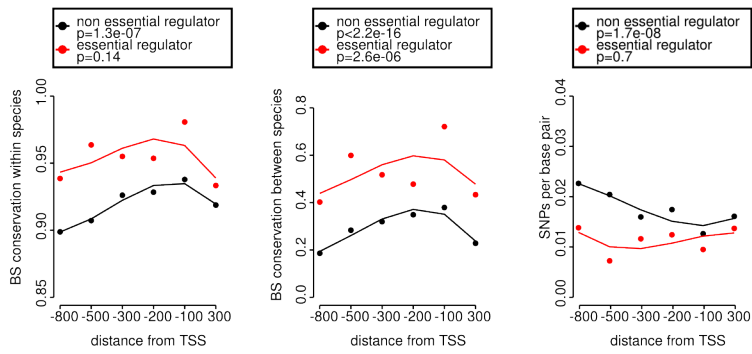

C

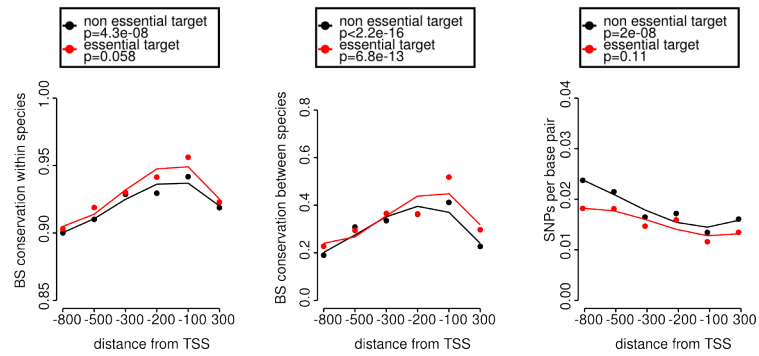

Supplement: Figure S6 — Binding sites closer to a transcription start site are under stronger selective constraint when controlling for potential confounders, including divergent promoters (A), essentiality of the regulator (B), and of the target gene (C). (PDF) [file pgen.1002077.s006.pdf]

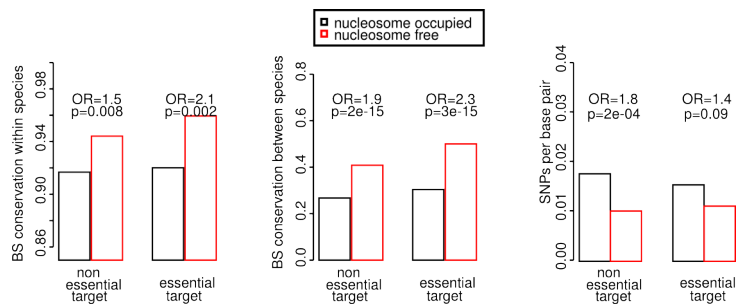

Supplement: Figure S7 — Binding sites in nucleosome free regions are more conserved also controlling for essentiality of the target gene. (PDF) [file pgen.1002077.s007.pdf]

A

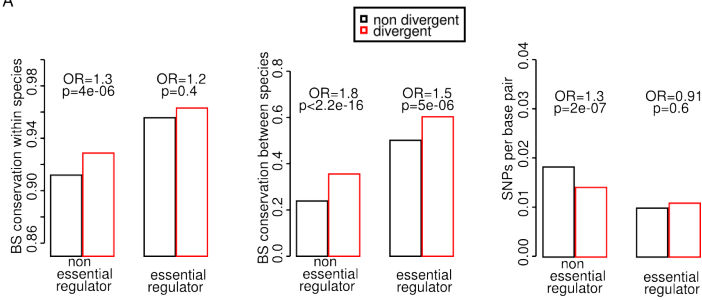

B

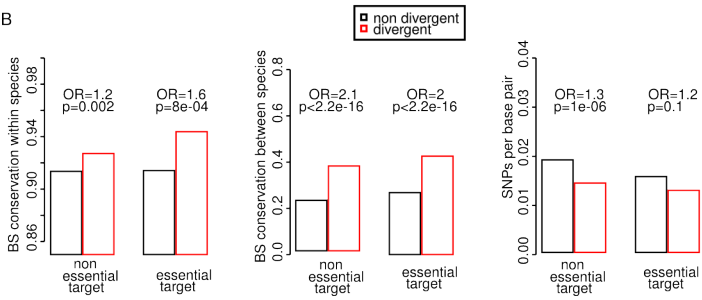

Supplement: Figure S8 — Binding sites in divergent promoters are more conserved even when accounting for target gene (A) and regulator essentiality (B). (PDF) [file pgen.1002077.s008.pdf]

A

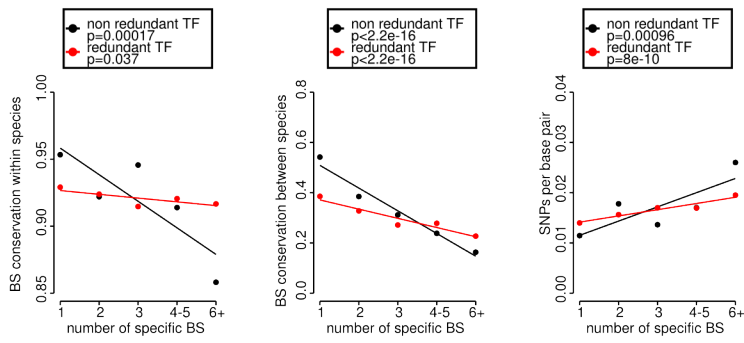

B

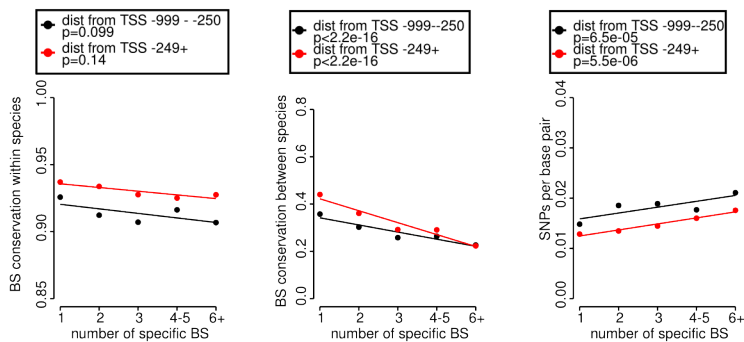

C

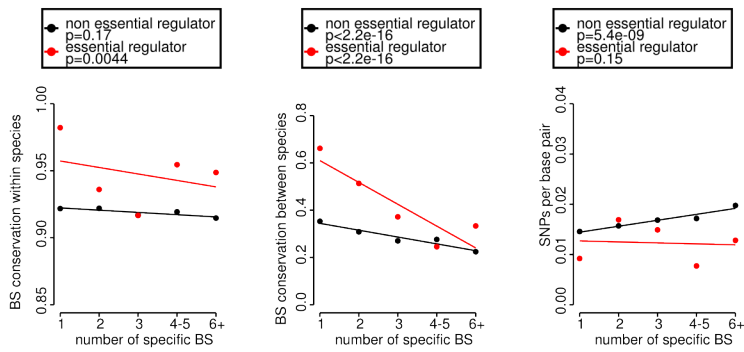

D

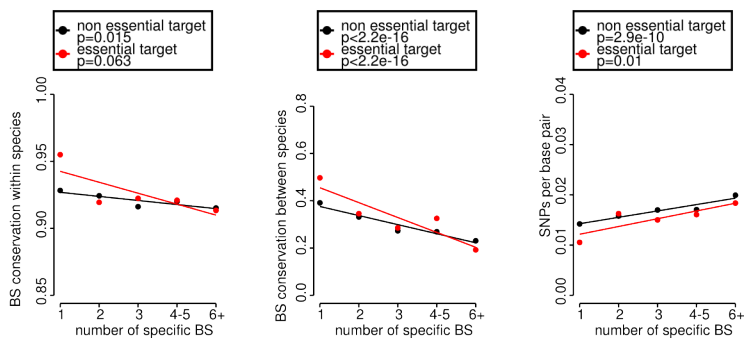

Supplement: Figure S9 — The potential for redundancy among binding sites relaxes constraints on individual binding sites. The plots show the fraction of conserved binding sites both between and within species (as defined in Materials and Methods) and the number of single nucleotide polymorphisms (SNPs) per base pair in binding sites within species. The effect of binding site redundancy is higher at a lower total number of binding sites in the promoters (A). Binding site redundancy relaxes constraint on binding sites at different distances from the transcription start site (B). The association between redundancy and reduced constraint is upheld when considering essential regulators (C) and essential target genes (D). (PDF) [file pgen.1002077.s009.pdf]

A

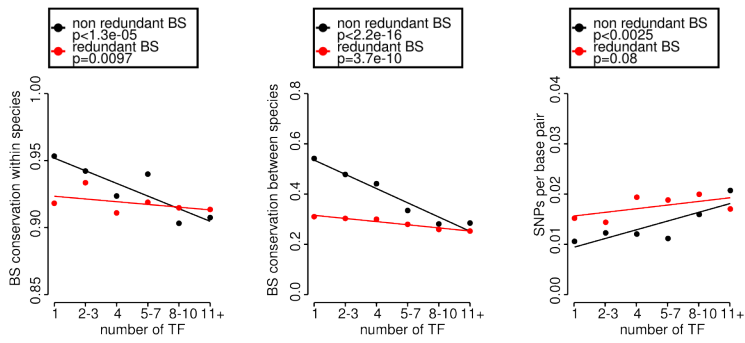

B

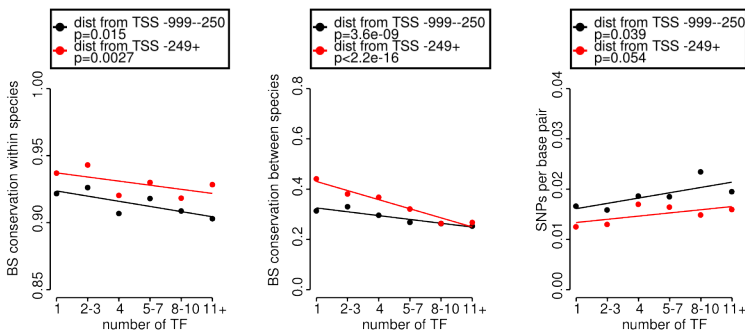

C

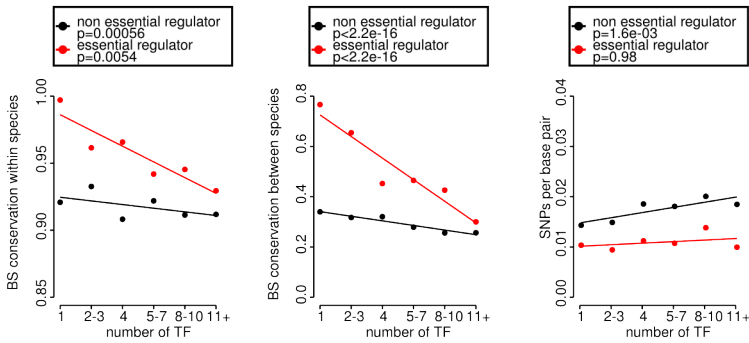

D

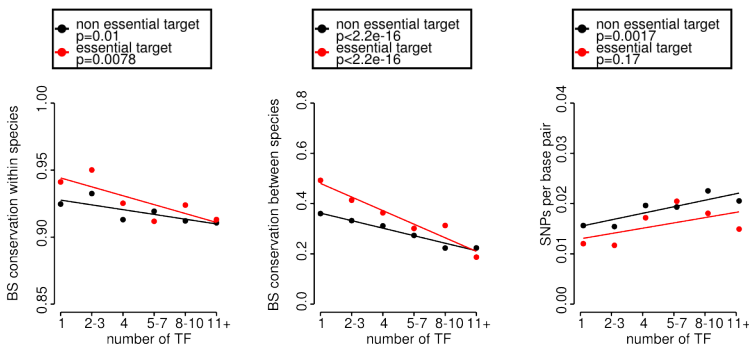

Supplement: Figure S10 — The potential for redundancy among transcription factors relaxes evolutionary constraints on TF binding sites. The plots show the fraction of conserved binding sites both between and within species (as defined in Materials and Methods) and the number of single nucleotide polymorphisms (SNPs) per base pair in binding sites within species. The association with transcription factor redundancy is stronger for non-redundant binding sites but it is also present for potentially redundant ones (A). Controlling for distance from the start sites (B), essentiality of the regulator (C) and essentiality of the target gene (D) also upholds the result. (PDF) [file pgen.1002077.s010.pdf]

A

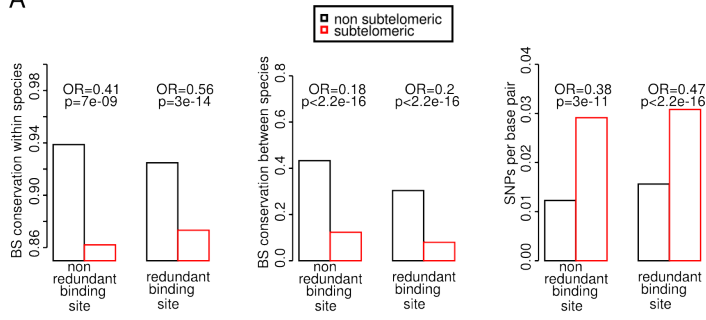

B

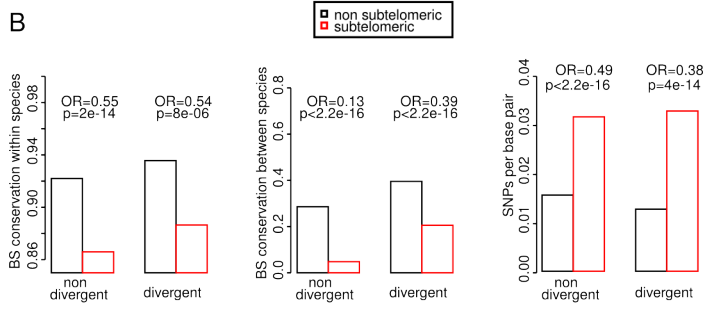

C

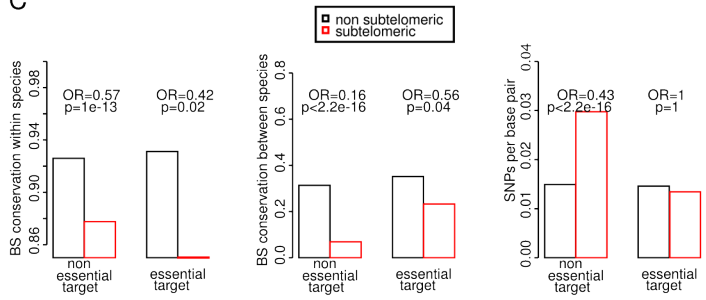

D

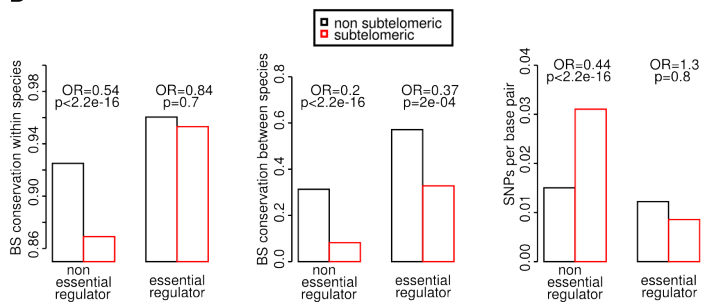

Supplement: Figure S11 — The reduced conservation of binding sites in sub-telomeric regions. The plots show the fraction of conserved binding sites both between and within species (as defined in Materials and Methods) and the number of single nucleotide polymorphisms (SNPs) per base pair in binding sites within species. The reduced conservation of binding sites in sub-telomeric regions is upheld when controlling for confounders such as binding site redundancy (A), divergent promoters (B), regulator (C), and target essentiality (D). (PDF) [file pgen.1002077.s011.pdf]

A

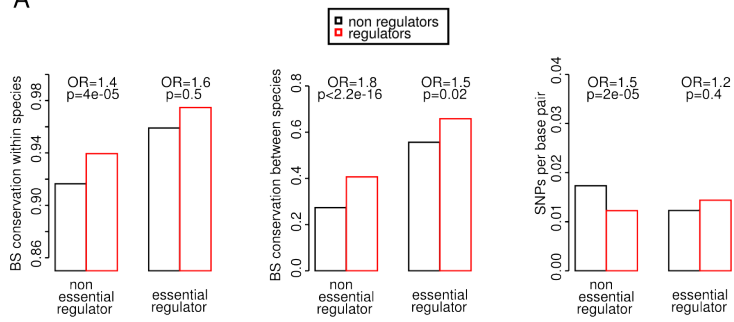

B

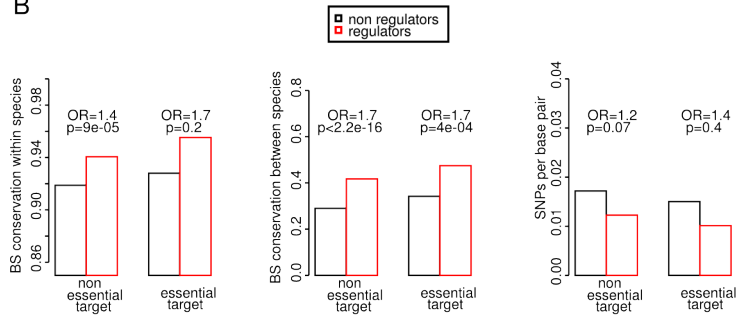

C

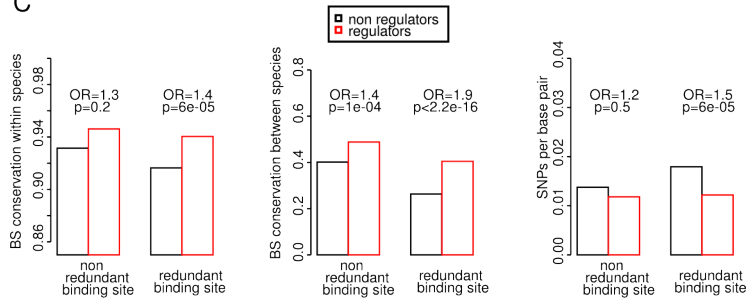

D

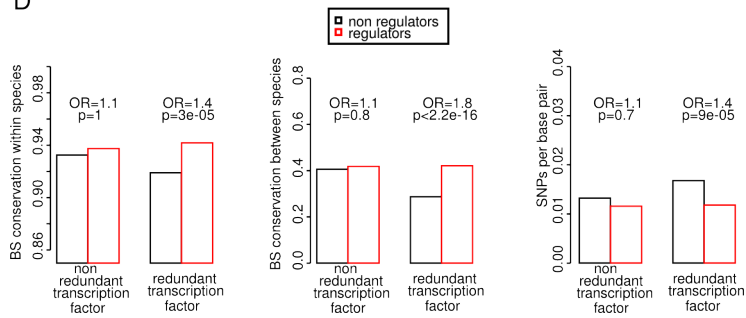

Supplement: Figure S12 — The increased constraint on binding sites in the promoters of regulatory genes is upheld when controlling to possible confounders such as the essentiality of the regulator (A), and redundancy among binding sites (B). The association is stronger when more than one TF targets the promoter (C), and upheld when controlling for the distance to a start site (D). (PDF) [file pgen.1002077.s012.pdf]

A

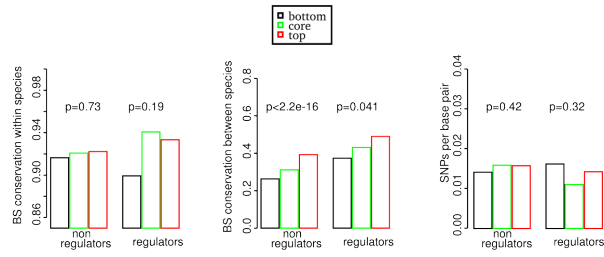

B

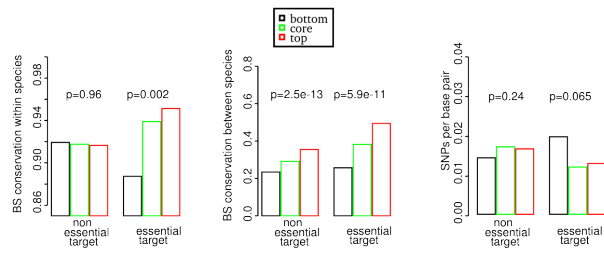

C

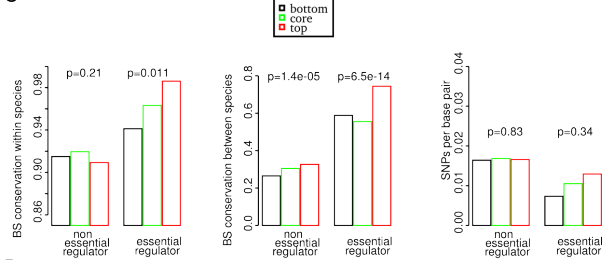

D

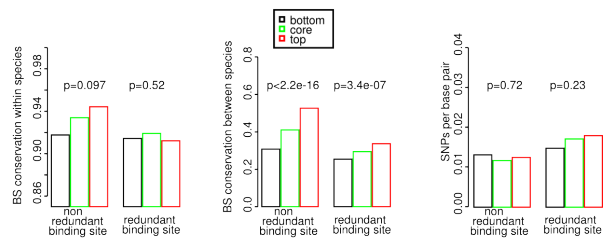

E

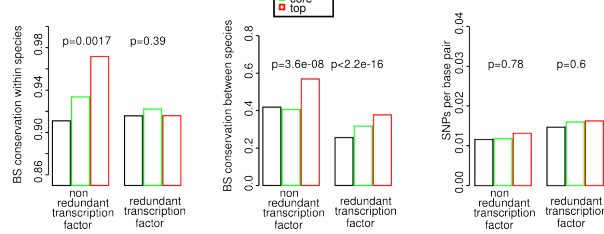

Supplement: Figure S13 — Binding sites for TFs higher in the regulatory hierarchy are more constrained when controlling for possible confounders such as identity of a target as a regulator (A), target importance (B), regulator importance (C), binding site redundancy (D), and the number of TFs that target a promoter (E). Indeed the association is stronger when controlling for target or regulator importance and for non-redundant binding sites. (PDF) [file pgen.1002077.s013.pdf]

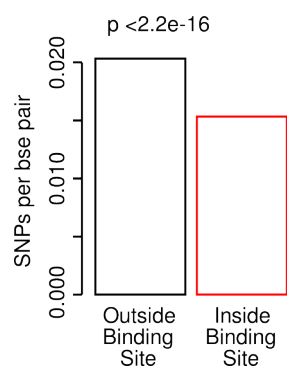

Supplement: Figure S14 — Nucleotide conservation compared inside binding sites to that in the 10 nucleotides downstream of each site (excluding nucleotides located within a known binding site). (PDF) [file pgen.1002077.s014.pdf]

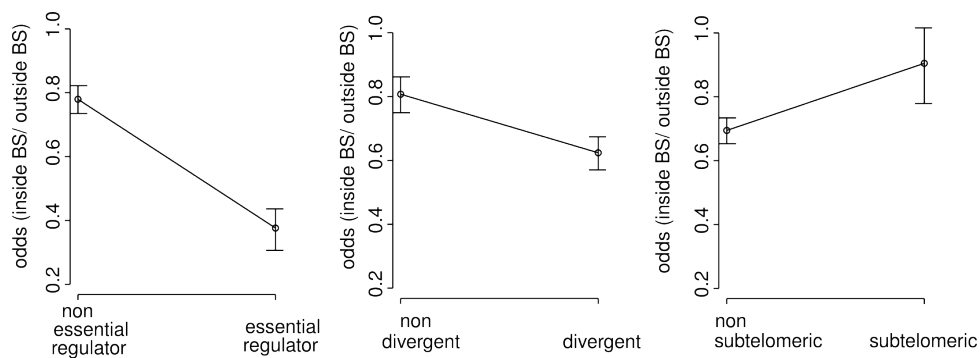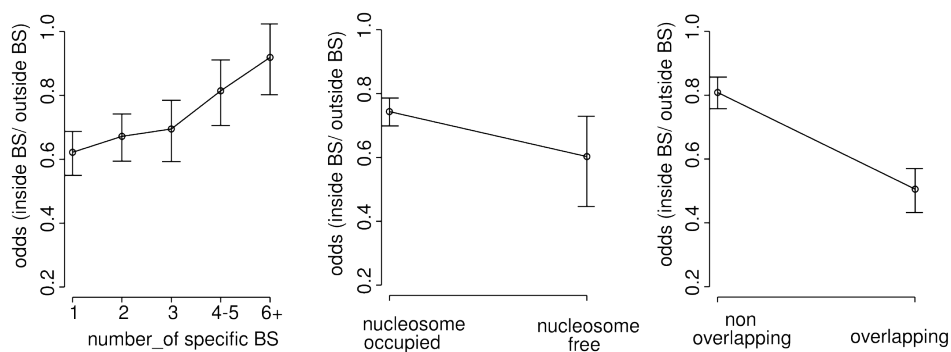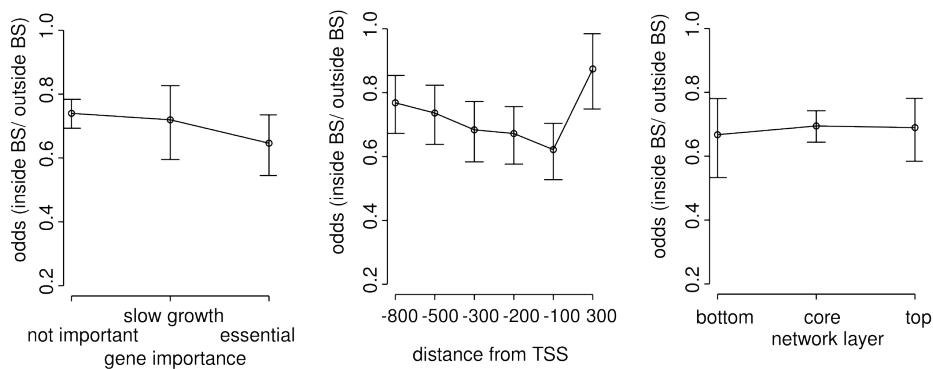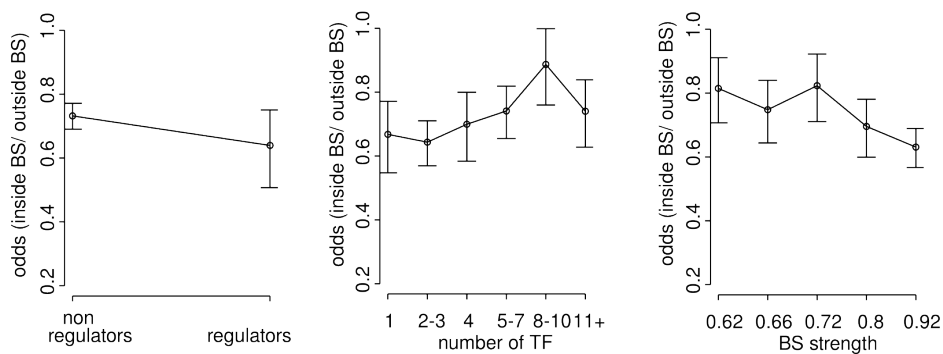

Supplement: Figure S15 — Comparison of the number of sequence changes per base pair within binding sites (BS) to that in the 10 bp downstream of each site (excluding bases located within known binding sites) for various properties. (PDF) [file pgen.1002077.s015.pdf]

A

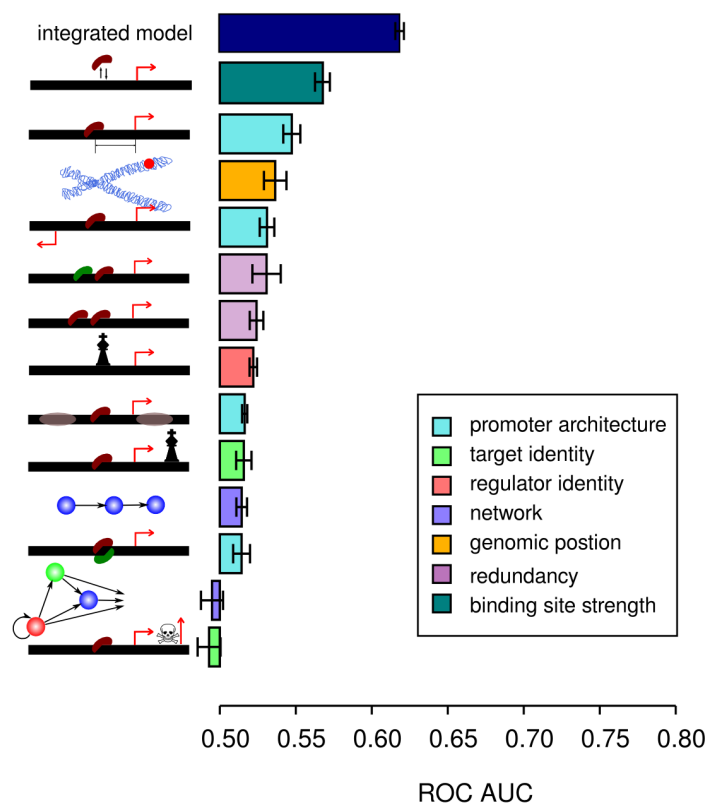

B

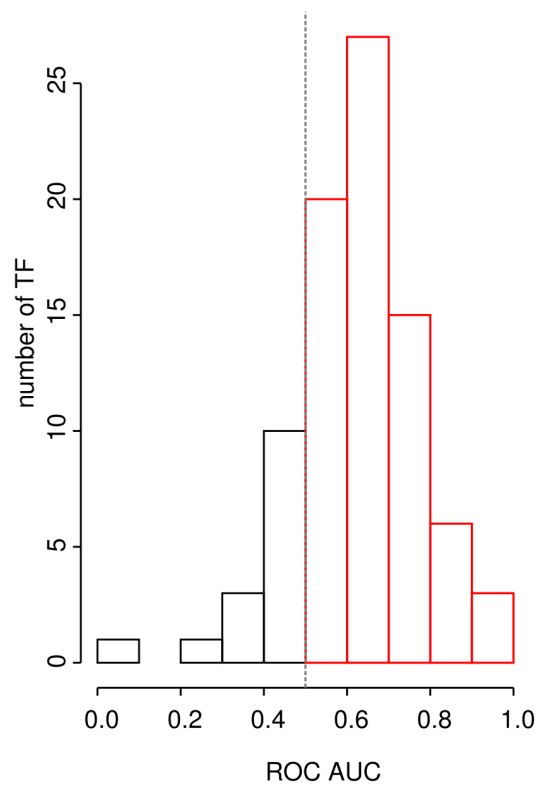

Supplement: Figure S16 — Predicting binding site conservation within species gives a similar qualitative relative performance as predicting conservation between species (see Figure 7), although the predictive power, as expected, is generally lower. The model shown was trained on within species conservation data and used to predict within species conservation (see Materials and Methods for further details). The predictive power is measured by the area under a receiver operating characteristic curve (ROC AUC, see Materials and Methods). (PDF) [file pgen.1002077.s016.pdf]

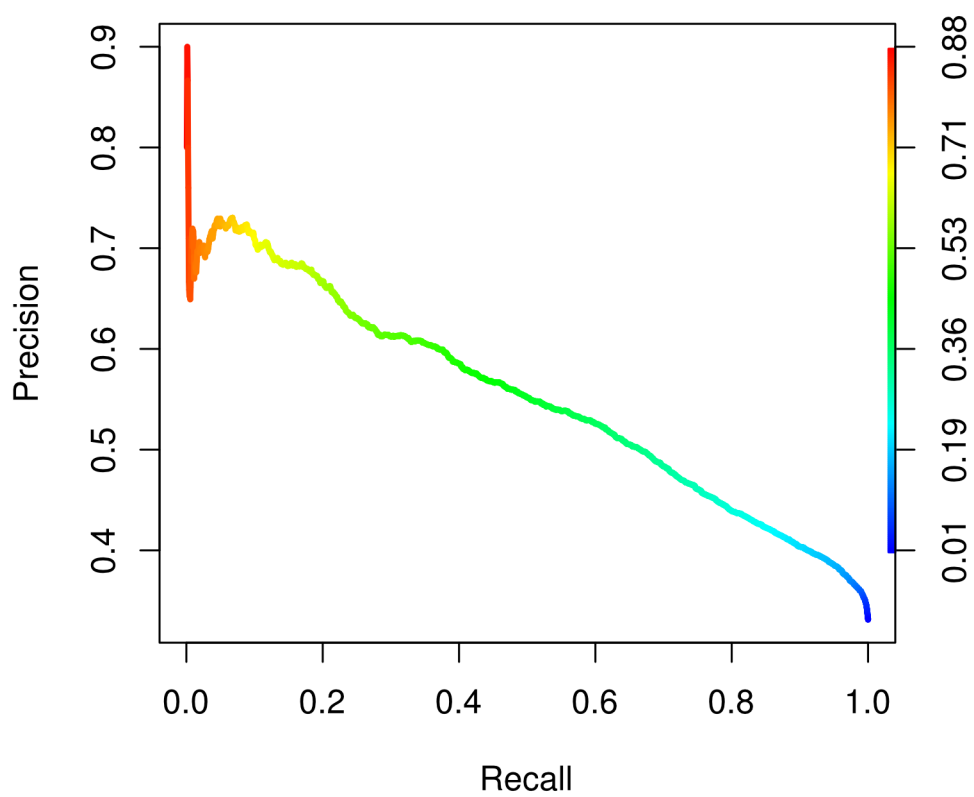

Supplement: Figure S17 — Precision-Recall plot showing the performance of the integrated model in predicting binding site conservation between species. (PDF) [file pgen.1002077.s017.pdf]

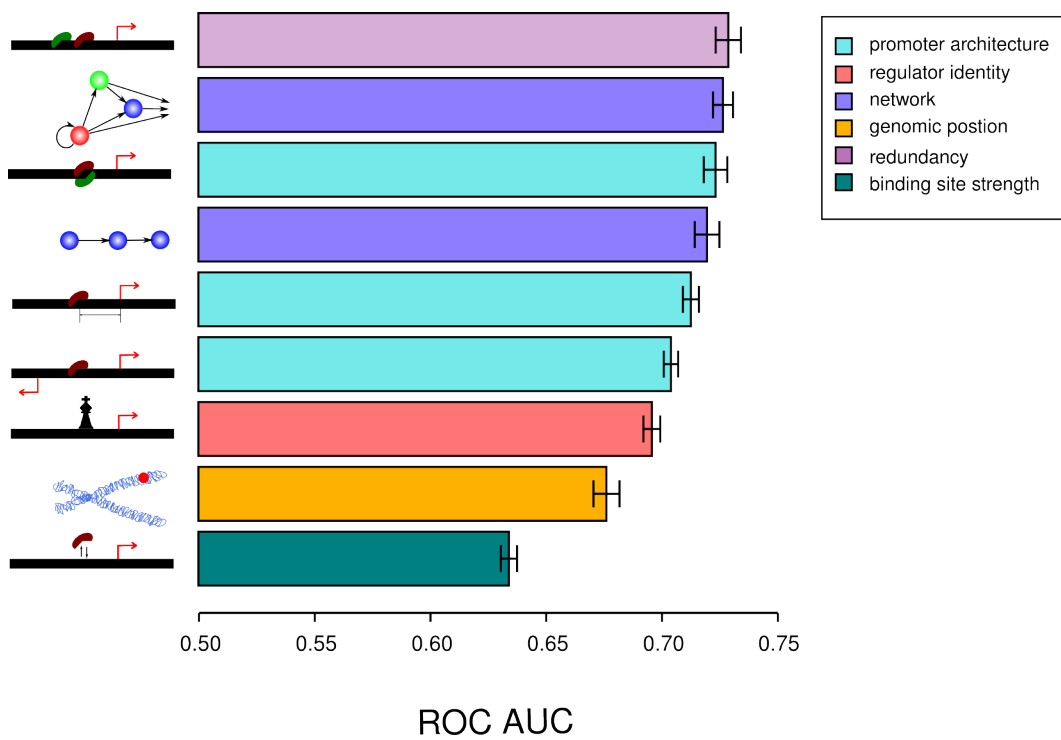

Supplement: Figure S18 — Predictive performance of the model for predicting the between species conservation of binding sites, evaluated after the addition of each additional variable. The predictive power is measured by the area under a receiver operating characteristic curve (ROC AUC). The mean and standard error of the AUC is shown for each model of increasing complexity. (PDF) [file pgen.1002077.s018.pdf]

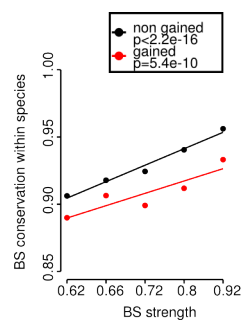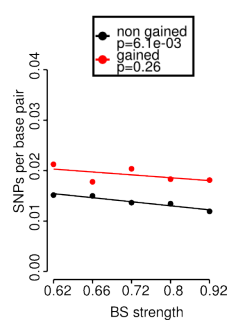

Supplement: Figure S19 — Stronger binding sites are more conserved when controlling for the gain of new binding sites (turnover). Stronger binding sites are less likely to arise de novo from the genomic background with the result that their stronger conservation could partially reflect a lower probability of compensation. To test this we scanned the promoters of the different S. cerevisiae strains and annotated when a new instance of a TF appeared in a promoter. We then analyzed the relationship between BS strength and BS conservation within species in the presence or absence of an alternative (gained) BS in the promoter of at least one of the strains. This shows that stronger binding sites are more conserved, even when taking into account the potential for compensation. (PDF) [file pgen.1002077.s019.pdf]
