# Supplementary material for: Integrated Genome-Scale Prediction of Detrimental Mutations in Transcription Networks
Source: PLoS Genet. 2011 May 26;7(5):e1002077. doi: 10.1371/journal.pgen.1002077 (PMC3102745; doi:10.1371/journal.pgen.1002077)
Supplement: Table S1 — Considering a subset of 24 TFs with in vitro confirmed binding site preferences supports the reported associations. Effects and significance are quantified using a generalized linear model (see Materials and Methods for further details). For categorical variables effect is the change in log odds of binding site conservation or base pair changes between the two categories, while for linear fits of discrete variables the effect is the change in log odds of conservation per unit of the variable. In this case distance from the transcription start site has been modeled with a linear fit up to the transcription start site instead of an orthogonal polynomial fit to simplify the comparison of the coefficients among the different datasets. Note that in the SNPs per base pair analysis the sign of the effect is the opposite to the within species and between species conservation because higher number of SNPs equates to a lower conservation. BS – binding site. (DOC) [file pgen.1002077.s020.doc]

**Table S1.** Considering a subset of 24 TFs with *in vitro* confirmed binding site preferences supports the reported associations. Effects and significance are quantified using a generalized linear model (see methods for further details). For categorical variables effect is the change in log odds of binding site conservation or base pair changes between the two categories, while for linear fits of discrete variables the effect is the change in log odds of conservation per unit of the variable. In this case distance from the transcription start site has been modeled with a linear fit up to the transcription start site instead of an orthogonal polynomial fit to simplify the comparison of the coefficients among the different datasets. Note that in the SNPs per base pair analysis the sign of the effect is the opposite to the within species and between species conservation because higher number of SNPs equates to a lower conservation. BS – binding site.

|  | all TFs | | | | | | *In vitro* confirmed TFs | | | | | |
| --- | --- | --- | --- | --- | --- | --- | --- | --- | --- | --- | --- | --- |
|  | Within species | | Between species | | SNPs per base pair | | Within species | | Between species | | SNPs per base pair | |
|  | effect | P value | effect | P value | effect | P value | effect | P value | effect | P value | effect | P value |
| Divergent promoters | 0.26 | 7.70E-008 | 0.61 | 1.20E-108 | -0.26 | 8.30E-008 | 0.5 | 1.50E-005 | 0.8 | 7.60E-044 | -0.43 | 1.5E-004 |
| Overlapping binding sites | 0.12 | 0.02 | 0.39 | 2.80E-045 | 0.06 | 3.10E-001 | 0.14 | 0.22 | 0.19 | 0.001 | 0.09 | 0.4 |
| Regulator targets | 0.33 | 8.90E-005 | 0.54 | 1.50E-003 | -0.323 | 2.60E-004 | 0.03 | 0.93 | 0.4 | 9.10E-006 | -0.25 | 0.22 |
| Essential regulator | 0.75 | 3.50E-011 | 1.16 | 3.50E-139 | -0.45 | 1.00E-007 | 0.54 | 0.02 | 0.53 | 2.80E-008 | -0.52 | 0.04 |
| Essential target | 0.15 | 0.04 | 0.33 | 1.90E-016 | -0.2 | 4.70E-003 | 0.43 | 0.03 | 0.5 | 2.70E-009 | -0.46 | 0.01 |
| Slow growth  target | 0.21 | 0.01 | 0.37 | 1.30E-001 | -0.19 | 0.02 | 0.24 | 0.18 | 0.31 | 3.90E-004 | -0.57 | 0.01 |
| Overexpression target | 0.03 | 0.63 | 0.35 | 1.30E-019 | -0.1 | 0.16 | 0.41 | 0.02 | 0.28 | 4.00E-004 | -0.36 | 0.05 |
| Nucleosome free BS | 0.59 | 1.20E-006 | 0.7 | 8.00E-041 | -0.48 | 4.30E-006 | 0.66 | 0.02 | 0.69 | 2.00E-010 | -0.6 | 0.02 |
| Subtelomeric regions BS | -0.65 | 7.50E-024 | -1.65 | 8.10E-120 | 0.83 | 2.50E-043 | -0.79 | 4.50E-008 | -1.44 | 8.70E-035 | 1.15 | 2.20E-019 |
| Transcription network hierarchy | 0.08 | 0.28 | 0.43 | 3.50E-024 | 0.07 | 0.39 | 0.31 | 0.11 | -0.06 | 0.59 | -0.08 | 0.79 |
| BS distance  from TSS | 5.20E-004 | 3.50E-009 | 6.10E-004 | 9.20E-027 | -5.17E-004 | 1.70E-009 | 3.60E-004 | 0.11 | 6.98E-004 | 9.60E-009 | -4.09E-004 | 5.00E-002 |
| Number of TFs | -0.02 | 1.50E-006 | -0.05 | 2.70E-048 | 0.02 | 1.50E-005 | -0.05 | 2.00E-005 | -0.1 | 2.00E-040 | 0.06 | 5.50E-006 |
| Number of  specific BS | -0.03 | 2.70E-003 | -0.11 | 3.90E-076 | 0.05 | 2.20E-010 | -0.1 | 0.01 | -0.12 | 4.70E-008 | 0.12 | 0.01 |
| BS strength | 1.91 | 3.30E-025 | 3.15 | 3.30E-214 | -0.54 | 2.80E-003 | 3.43 | 4.40E-012 | 4.66 | 2.00E-087 | -0.83 | 0.06 |
