# Supplementary material for: Integrated Genome-Scale Prediction of Detrimental Mutations in Transcription Networks
Source: PLoS Genet. 2011 May 26;7(5):e1002077. doi: 10.1371/journal.pgen.1002077 (PMC3102745; doi:10.1371/journal.pgen.1002077)
Supplement: Table S2 — Most of the associations reported in this manuscript are also observed for a majority of individual TFs when examined in isolation. The proportion of individual TFs showing the reported effect are shown, also when restricting to TFs that show an effect significant at the 5% level. TSS – transcription start site. (DOC) [file pgen.1002077.s021.doc]

**Table S2.** Most of the associations reported in this manuscript are also observed for a majority of individual TFs when examined in isolation. The proportion of individual TFs showing the reported effect are shown, also when restricting to TFs that show an effect significant at the 5% level. TSS – transcription start site.

|  | Between species | Between species (p<0.05) | Within species | Within species (p<0.05) | Number of SNPs | Number of SNPs (p<0.05) |
| --- | --- | --- | --- | --- | --- | --- |
| Divergent promoters | 0.81 | 0.94 | 0.63 | 0.85 | 0.62 | 0.9 |
| Overlapping binding sites | 0.68 | 0.83 | 0.66 | 0.67 | 0.53 | 0.38 |
| Regulator target | 0.67 | 0.89 | 0.75 | 0.82 | 0.76 | 0.7 |
| Essential target | 0.59 | 0.79 | 0.61 | 0.50 | 0.67 | 0.6 |
| Overexpression target | 0.66 | 0.85 | 0.52 | 0.50 | 0.55 | 0.6 |
| Nucleosome free BS | 0.64 | 0.95 | 0.71 | 0.75 | 0.70 | 1 |
| Slow growth target | 0.71 | 0.74 | 0.64 | 0.67 | 0.62 | 0.8 |
| Subtelomeric BS | 0.85 | 0.96 | 0.59 | 0.94 | 0.67 | 1 |
| BS distance from TSS | 0.81 | 0.90 | 0.65 | 0.60 | 0.57 | 0.9 |
| Number of TF | 0.68 | 0.82 | 0.52 | 0.69 | 0.55 | 0.69 |
| Number of specific BS | 0.57 | 0.49 | 0.41 | 0.46 | 0.48 | 0.46 |
| BS strength | 0.68 | 0.83 | 0.66 | 0.67 | 0.55 | 0.69 |
