# Supplementary material for: Integrated Genome-Scale Prediction of Detrimental Mutations in Transcription Networks
Source: PLoS Genet. 2011 May 26;7(5):e1002077. doi: 10.1371/journal.pgen.1002077 (PMC3102745; doi:10.1371/journal.pgen.1002077)
Supplement: Table S3 — Transcription factors whose binding sites are more conserved when present in multiple copies in a promoter have weaker binding sites and more binding sites in each promoter. Wilcoxon test p-values on testing the hypothesis that the selected transcription factors have lower binding site strength and higher number of binding sites compared to transcription factors are shown for all transcription factors that show a significant (p<0.05) positive relationship between conservation and the number of binding sites in the promoter are selected. (DOC) [file pgen.1002077.s022.doc]

**Table S3.** Transcription factors whose binding sites are more conserved when present in multiple copies in a promoter have weaker binding sites and more binding sites in each promoter. Wilcoxon test p-values on testing the hypothesis that the selected transcription factors have lower binding site strength and higher number of binding sites compared to transcription factors are shown for all transcription factors that show a significant (p<0.05) positive relationship between conservation and the number of binding sites in the promoter are selected.

|  | Binding site strength  p-value | Number of specific binding sites p-value |
| --- | --- | --- |
| Between species | <2.2E-16 | 8.22E-013 |
| Within species | 1 | < 2.2E-16 |
| Number of SNPs | < 2.2E-16 | < 2.2E-16 |
