# Supplementary material for: Integrated Genome-Scale Prediction of Detrimental Mutations in Transcription Networks
Source: PLoS Genet. 2011 May 26;7(5):e1002077. doi: 10.1371/journal.pgen.1002077 (PMC3102745; doi:10.1371/journal.pgen.1002077)
Supplement: Table S4 — Summary of the fit of the integrated model for binding site conservation between species. The model was fitted using a generalized linear model framework (see Materials and Methods for further details). For distance from TSS a third degree orthogonal polynomial fit was used (see method). Hierarchy of the regulator was modeled as an ordered factor with two levels and orthogonal polynomial contrasts have been fitted. The estimated effects for categorical variables represent the log odds of the conservation between two categories, while for linear fit of discrete variables they represent the log odds per unit of variable. The table also shows coefficient, standard errors, the z-values and the p-values. The BS strength (score) is the most important determinant of binding site conservation but the other determinants still independently explain part of binding site conservation. (DOC) [file pgen.1002077.s023.doc]

**Table S4.** Summary of the fit of the integrated model for binding site conservation between species. The model was fitted using a generalized linear model framework (see methods for further details). For distance from TSS a third degree orthogonal polynomial fit was used (see method). Hierarchy of the regulator was modeled as an ordered factor with two levels and orthogonal polynomial contrasts have been fitted. The estimated effects for categorical variables represent the log odds of the conservation between two categories, while for linear fit of discrete variables they represent the log odds per unit of variable. The table also shows coefficient, standard errors, the z-values and the p-values. The BS strength (score) is the most important determinant of binding site conservation but the other determinants still independently explain part of binding site conservation.

|  | Estimated  effect | Std. Error | z value | P-value (>|z|) |
| --- | --- | --- | --- | --- |
| Intercept | -3.89 | 0.11 | -35.9 | <2.2E-16 |
| Distance from TSS (linear) | 22.65 | 2.97 | 7.61 | 2.65E-014 |
| Distance from TSS (quadratic) | -34.35 | 3.02 | -11.36 | <2.2E-16 |
| Distance from TSS (cubic) | -6.65 | 3.22 | -2.06 | 0.04 |
| Number of specific BS | -0.02 | 0.01 | -2.65 | 0.01 |
| Divergent promoter | 0.48 | 0.03 | 14.59 | <2.2E-16 |
| BS strength | 3.53 | 0.12 | 28.71 | <2.2E-16 |
| Overlapping BS | 0.27 | 0.03 | 8.06 | 7.38E-016 |
| Essential regulator | 0.93 | 0.06 | 16.69 | <2.2E-16 |
| Sub-telomeric location | -1.6 | 0.08 | -20.25 | <2.2E-16 |
| Hierarchy (linear) | 0.47 | 0.05 | 10.08 | <2.2E-16 |
| Hierarchy (quadratic) | -0.06 | 0.03 | -1.92 | 0.05 |
| Regulator target | 0.61 | 0.05 | 12.08 | <2.2E-16 |
