# Supplementary material for: Integrated Genome-Scale Prediction of Detrimental Mutations in Transcription Networks
Source: PLoS Genet. 2011 May 26;7(5):e1002077. doi: 10.1371/journal.pgen.1002077 (PMC3102745; doi:10.1371/journal.pgen.1002077)
Supplement: Table S5 — Comparison between the final model and simpler models that exclude each of the analysed features. The analysis of deviance tables show degrees of freedom, residual unexplained deviance, the likelihood ratio test and its p-value. Binding site strength is the feature that causes the highest decrease in explanatory power (increase in residual deviance) when excluded from the model. Each of the features contributes significantly to the explanatory power of the model. (DOC) [file pgen.1002077.s024.doc]

**Table S5.** Comparison between the final model and simpler models that exclude each of the analysed features. The analysis of deviance tables show degrees of freedom, residual unexplained deviance, the likelihood ratio test and its p-value. Binding site strength is the feature that causes the highest decrease in explanatory power (increase in residual deviance) when excluded from the model. Each of the features contributes significantly to the explanatory power of the model

|  | Degrees of freedom | Residual Deviance | Likelihood Ratio Test | P-value (Chi square) |
| --- | --- | --- | --- | --- |
| Number of TF | 1 | 22284.44 | 71.02 | 3.54E-017 |
| Hierarchy | 2 | 22316.6 | 103.18 | 3.93E-023 |
| Overlapping BS | 1 | 22319.3 | 105.88 | 7.83E-025 |
| Regulator target | 1 | 22367.84 | 154.42 | 1.88E-035 |
| Distance from TSS | 3 | 22386.53 | 173.11 | 2.71E-037 |
| Divergent promoter | 1 | 22396.45 | 183.03 | 1.06E-041 |
| Essential regulator | 1 | 22479.63 | 266.21 | 7.62E-060 |
| Subtelomeric | 1 | 22773.39 | 559.96 | 8.56E-124 |
| BS strength | 1 | 23043.38 | 829.95 | 1.66E-182 |
