# Supplementary material for: Integrated Genome-Scale Prediction of Detrimental Mutations in Transcription Networks
Source: PLoS Genet. 2011 May 26;7(5):e1002077. doi: 10.1371/journal.pgen.1002077 (PMC3102745; doi:10.1371/journal.pgen.1002077)
Supplement: Table S6 — Binding sites experimentally validated as important for gene expression or fitness. The list includes binding sites from the systematic dataset supported by an independent experimental report. Each BS has been mutated or deleted and a deleterious effect on the expression of the neighboring gene or fitness verified. (DOC) [file pgen.1002077.s025.doc]

**Table S6.** Binding sites experimentally validated as important for gene expression or fitness. The list includes binding sites from the systematic dataset supported by an independent experimental report. Each BS has been mutated or deleted and a deleterious effect on the expression of the neighboring gene or fitness verified.

| Gene | TF | Chr | BS start | BS end | References |
| --- | --- | --- | --- | --- | --- |
| YAL005C | HSF1 | chr01 | 156659 | 156674 | Mol. Cell. Biol. 13:5637-5646 (1993)/ Mol. Cell. Biol. Park and Craig 9 (5): 2025 |
| YBR093C | PHO4 | chr02 | 450748 | 450755 | Nucleic Acids Res 1996, 24:4479-4486/ Mol. Cell. Biol. 9:2050-2057 (1989)/ Nucleic Acids Res 1992, 20:1031-1038 |
| YBR248C | GCN4 | chr02 | 751298 | 751307 | J Biol Chem 1996, 271:29637-29643 |
| YBR248C | GCN4 | chr02 | 751384 | 751393 | J Biol Chem 1996, 271:29637-29643 |
| YCL030C | GCN4 | chr03 | 71248 | 71257 | Mol Cell Biol 1991, 11:3642-3651 |
| YCL030C | RAP1 | chr03 | 71258 | 71269 | Mol Cell Biol 1991, 11:3642-3651 |
| YDR146C | FKH2 | chr04 | 794991 | 795004 | Mol Cell Biol, 1995, 15:5917-5928 |
| YDR146C | NDD1 | chr04 | 794994 | 795005 | Mol Cell Biol, 1995, 15:5917-5928 |
| YDR146C | MCM1 | chr04 | 795002 | 795019 | Mol Cell Biol, 1995, 15:5917-5928 |
| YDR354W | GCN4 | chr04 | 1251434 | 1251443 | EMBO J. 9:2951-2957 (1990) |
| YER111C | MCM1 | chr05 | 411783 | 411800 | Genes Dev, 1997, 11:1277-1288 |
| YFL026W | MCM1 | chr06 | 93116 | 93133 | Genes Dev 1991, 5:2405-2419/ Cell. 1987 Jul 31;50(3):369-77 |
| YGL234W | BAS1 | chr07 | 61888 | 61896 | PNAS 1992, 89:6746-6750 |
| YGL234W | BAS1 | chr07 | 61921 | 61929 | PNAS 1992, 89:6746-6750 |
| YGR059W | ABF1 | chr07 | 660207 | 660221 | Mol Cell Biol 1997, 17:1152-1159 |
| YHR037W | PUT3 | chr08 | 191486 | 191501 | Mol Cell Biol 1989, 9:4706-4712 |
| YJL106W | UME6 | chr10 | 240426 | 240437 | Mol Cell Biol, 1997, 17:3536-3546 |
| YJR048W | HAP1 | chr10 | 574177 | 574188 | Nucleic Acids Res 1996, 24:1453-1459 |
| YKL209C | MCM1 | chr11 | 48532 | 48549 | J Biol Chem 1997, 272:8402-8409/ Genes Dev. 3:921-935 (1989) |
| YKL182W | RAP1 | chr11 | 121899 | 121910 | Eur J Biochem 1994, 225:213-222 |
| YKL182W | ABF1 | chr11 | 121952 | 121966 | Eur J Biochem 1994, 225:213-222 |
| YLR214W | MAC1 | chr12 | 591275 | 591286 | J Biol Chem 1997, 272:15951-15958 |
| YLR214W | MAC1 | chr12 | 591292 | 591304 | J Biol Chem 1997, 272:15951-15958 |
| YLR286C | ACE2 | chr12 | 755176 | 755185 | Mol Cell Biol 1996, 16:1746-1758 |
| YLR286C | ACE2 | chr12 | 755196 | 755205 | Mol Cell Biol 1996, 16:1746-1758 |
| YML054C | HAP1 | chr13 | 181861 | 181872 | Mol Cell Biol 1991, 11:3762-3772 |
| YMR186W | REB1 | chr13 | 678955 | 678964 | Nucleic Acids Res 1995, 23:1822-1829/ Mol Cell Biol 1996, 16:7004-7017 |
| YMR199W | MBP1 | chr13 | 708977 | 708985 | J Biol Chem 1997, 272:9071-9077 |
| YMR199W | SWI6 | chr13 | 708978 | 708985 | J Biol Chem 1997, 272:9071-9077 |
| YNL102W | MBP1 | chr14 | 471873 | 471881 | Proc. Natl. Acad. Sci. USA 88:7155-7159 (1991) |
| YNL102W | MBP1 | chr14 | 471908 | 471916 | Proc. Natl. Acad. Sci. USA 88:7155-7159 (1991) |
| YOR065W | CBF1 | chr15 | 477243 | 477252 | Nucleic Acids Res 1996, 24:2395-2403 |
| YOR065W | HAP1 | chr15 | 477292 | 477303 | Mol. Gen. Genet. 232, 447–459/Nucleic Acids Res 1996, 24:1453-1459 / Mol. Cell. Biol. 11:4934-4942 (1991) |
| YOR120W | GAL4 | chr15 | 585282 | 585301 | J Biol Chem 1997, 272:31630-31635/ Mol Gen Genet. 1997 Nov;256(6):682-9 |
| YOR128C | BAS1 | chr15 | 600880 | 600888 | PNAS 1992, 89:6746-6750 |
| YOR128C | BAS1 | chr15 | 600923 | 600931 | PNAS 1992, 89:6746-6750 |
| YOR202W | GCN4 | chr15 | 777331 | 777340 | Mol Cell Biol 1995, 15:7059-7066/ Science 234:451-457 (1986) |
| YPR119W | REB1 | chr16 | 818419 | 818428 | Nucleic Acids Res. 2003 August 1; 31(15): 4597–4607 |
| YPR119W | MCM1 | chr16 | 818515 | 818532 | Mol Cell Biol, 1994, 14:348-359 |
| YPR119W | NDD1 | chr16 | 818528 | 818539 | Mol Cell Biol, 1995, 15:5917-5928 |
| YPR119W | FKH2 | chr16 | 818529 | 818542 | Mol Cell Biol, 1995, 15:5917-5928 |
| YPR119W | FKH1 | chr16 | 818533 | 818542 | Mol Cell Biol, 1995, 15:5917-5928 |
| YPR124W | MAC1 | chr16 | 835082 | 835093 | J Biol Chem 1997, 272:15951-15 |
| YPR124W | MAC1 | chr16 | 835106 | 835117 | J Biol Chem 1997, 272:15951-15 |
